# TXNIP mediates high glucose-induced mitophagic flux and lysosome enlargement in human retinal pigment epithelial cells

**DOI:** 10.1242/bio.038521

**Published:** 2019-04-25

**Authors:** Takhellambam S. Devi, Thangal Yumnamcha, Fayi Yao, Mallika Somayajulu, Renu A. Kowluru, Lalit P. Singh

**Affiliations:** Department of Ophthalmology, Visual and Anatomical Sciences (OVAS), Wayne State University School of Medicine, Detroit, MI 48201, USA

**Keywords:** Hyperglycemia, TXNIP, Mitophagy, Lysosome destabilization, Retinal pigment epithelium

## Abstract

Thioredoxin-interacting protein (TXNIP) plays a critical role in oxidative stress, inflammation, apoptosis and the pathogenesis of diabetic retinopathy (DR). However, the role of TXNIP in high glucose-induced retinal pigment epithelium (RPE) dysfunction is still unknown. Here, we show that high glucose (HG; 25 mM,) significantly increases TXNIP expression at both the mRNA and protein levels when compared to low glucose (LG; 5.5 mM) in a human RPE cell line (ARPE-19) and primary human RPE (HRPE) cells. TXNIP upregulation is associated with mitochondrial membrane depolarization, fragmentation and mitophagic flux to lysosomes. We used confocal live-cell imaging of RPE cells expressing mt-Keima, a coral protein that emits green light in mitochondria (alkaline or neutral pH) and red light in the acidic lysosome, to measure mitophagic flux. We observed an elongated mitochondrial network of green mt-Keima under LG, which is fragmented in HG. Red mt-Keima accumulates in lysosomes as small punctate aggregations under LG in both ARPE-19 and HRPE cells, whereas they are significantly enlarged (two- to threefold) under HG. Lysosomal enlargement under HG is further illustrated by lysosomal membrane protein LAMP1-mCherry expression in both ARPE-19 and HRPE cells. Furthermore, HG causes lysosomal cathepsin L inactivation and pro-inflammatory caspase-1 activation in ARPE-19 cells. TXNIP knockdown by shRNA prevents mitochondrial fragmentation, mitophagic flux and lysosome enlargement under HG. In addition, antioxidant N-acetylcysteine (NAC) and Amlexanox (Amlx), an inhibitor of protein kinase TBK1 and of the mitophagic adaptors Optineurin (Optn) and Sequestosome 1 (p62/SQSTM1), prevent mitophagic flux and lysosome enlargement. These results suggest that TXNIP mediates several deleterious effects of high glucose on RPE, which may be implicated in the development of DR.

## Abstract (precis)

**Summary:** Mitophagy is critical for removing damaged mitochondria and maintaining cellular health. Here, the influence of high glucose and TXNIP on mitophagy-lysosome axis dysregulation in human retinal pigment epithelium is revealed.

## INTRODUCTION

Diabetic retinopathy (DR) is the number one cause of blindness among the working adult population around the globe, including the US. DR is primarily defined by microvascular complications of retinal blood vessels, including endothelial dysfunction, pericyte dropout, basement membrane thickening, acellular capillary formation, inner blood-retinal barrier breakdown, aneurysms and fragile new blood vessel formation (Roy et al., 2017; Wong et al., 2016). In addition, recent studies have also shown that retinal neurons are damaged in the early stages and that glial activation may play an important role in the pathogenesis of DR (Abcouwer and Gardner, 2014; Srinivasan et al., 2017). Photoreceptors are the largest cell population in the retina and they are responsible for visual perception by capturing photons with rhodopsin and cone opsin in their outer segments (Ponnalagu et al., 2017). In DR, photoreceptor dysfunction occurs early, before microvascular damage (Simó et al., 2018; Tonade et al., 2017). The retinal pigment epithelium (RPE) is a single layer of fully differentiated cells that separates the neuroretina from the fenestrated choriocapillaris that help form the outer blood-retinal barrier (Simó et al., 2010).

The apical plasma membrane of the RPE interacts with the photoreceptor outer segment (POS) and phagocytoses them daily to recycle retinoic acid pigments back to photoreceptors (Mazzoni et al., 2014). In addition, RPE supplies glucose and oxygen to photoreceptors, which are required to meet photoreceptors' high bioenergetic demand (Mazzoni et al., 2014; Simó et al., 2010). In addition, RPE contains melanin, which absorbs excess light, which is especially important in the macular region, where visual color perceptions are high with cone photoreceptors. Therefore, RPE dysfunction is associated with several photoreceptor degenerative diseases that lead to blindness, including retinitis pigmentosa and age-related macular degeneration (Keeling et al., 2018; Mittal et al., 2018); however, the influence of hyperglycemia on RPE dysfunction in diabetes and its contribution to DR has not been investigated fully. Because RPE is also lined by fenestrated capillaries at the Bruch's membrane, RPE receives glucose and oxygen directly from choriocapillaris (Mazzoni et al., 2014; Simó et al., 2010). RPE also stores glycogen and utilizes glycolysis for ATP generation in addition to mitochondrial oxidative phosphorylation, which generates reactive oxygen species (ROS) (Mazzoni et al., 2014; Simó et al., 2010). On the other hand, retinal neurons, including photoreceptors, use oxidative phosphorylation for their energy requirements. In fact, the POS has been shown to generate high levels of ROS in early DR (Du et al., 2013). These ROS may cause damage to both neurons and RPE if these ROS are not neutralized adequately. In fact, diabetes and chronic hyperglycemia-associated oxidative stress, low-grade inflammation, and premature cell death are known to play critical roles in the etiology of DR (Fu et al., 2018).

Thioredoxin-interacting protein, TXNIP, expression is strongly induced by diabetes and high glucose (HG) levels in all tissues examined, including different cell types in the retina (Cheng et al., 2006; Devi et al., 2012, 2013; Perrone et al., 2009, 2010; Singh and Perrone, 2013). TXNIP is considered a pro-oxidative stress, pro-inflammatory and pro-apoptotic protein that has been shown to play a critical role in retinal microvascular and neuronal injury in both *in vivo* and *in vitro* studies (Singh and Perrone, 2013). TXNIP binds to and inhibits the antioxidant and thiol-reducing capacity of thioredoxins, thereby causing cellular oxidative stress and apoptosis. Trx1 is present in the cytosol and nucleus, whereas Trx2 is the mitochondrial isoform. TXNIP also causes mitochondrial damage and mitophagy in retinal Müller cells under HG conditions in culture, and TXNIP knockdown by siRNA in diabetic retinas prevents autophagic LC3B puncta formation (Devi et al., 2017; Singh et al., 2017). Nonetheless, research on the role of TXNIP in mitophagy mechanisms and dysfunction in RPE under high-glucose conditions is still lacking. Therefore, we investigated the extent to which HG exposure causes mitochondrial damage and mitophagic flux to lysosomes in human RPE cells and the role, if any, that TXNIP plays in these processes.

Mitophagy is an autophagic process that removes damaged, old and/or dysfunctional mitochondria via lysosomal degradation (Devi et al., 2017; Singh et al., 2017; Zhou et al., 2011). Damaged mitochondria, if not removed, produce excess ROS and little ATP, activate the NLPR3 inflammasome and cause cell death (Zhou et al., 2011). Therefore, efficient mitophagy is required for cell survival and tissue protection. However, excess mitophagic flux may cause lysosome overloading, enlargement and destabilization, leading to lysosomal or autophagic cell death (Devi et al., 2017; Singh et al., 2017). In this study, we used an adenovirus encoding mt-Keima (Ad-CMV-mt-Keima construct), a coral-derived fluorophore protein targeted to mitochondria by tagging the mitochondrial target sequence of cytochrome C subunit VIII (Singh et al., 2017; Sun et al., 2017; Williams and Ding, 2018). Mt-Keima emits green light at alkaline or neutral pHs (e.g. the pH in the mitochondrion) and emits red at acidic pHs (e.g. inside the lysosome) after mitophagic flux. Furthermore, mt-Keima is resistant to lysosomal acid hydrolases and therefore accumulates for a longer period of time inside the lysosome. In addition, we also used a human LAMP1 construction in Ad-CMV-LAMP1-mCherry (Pickles et al., 2018) to measure lysosomal size and morphology directly in human RPE cells under HG conditions. Our results show that HG levels upregulate TXNIP significantly in RPE cells in culture, resulting in mitochondrial fragmentation, mitophagic flux and lysosomal enlargement/destabilization. TXNIP knockdown by shRNA prevents some of the deleterious effects of HG levels on RPE cells.

## RESULTS

### HG levels induce TXNIP expression and mitochondrial dysfunction in RPE cells

**Fig. 1.**
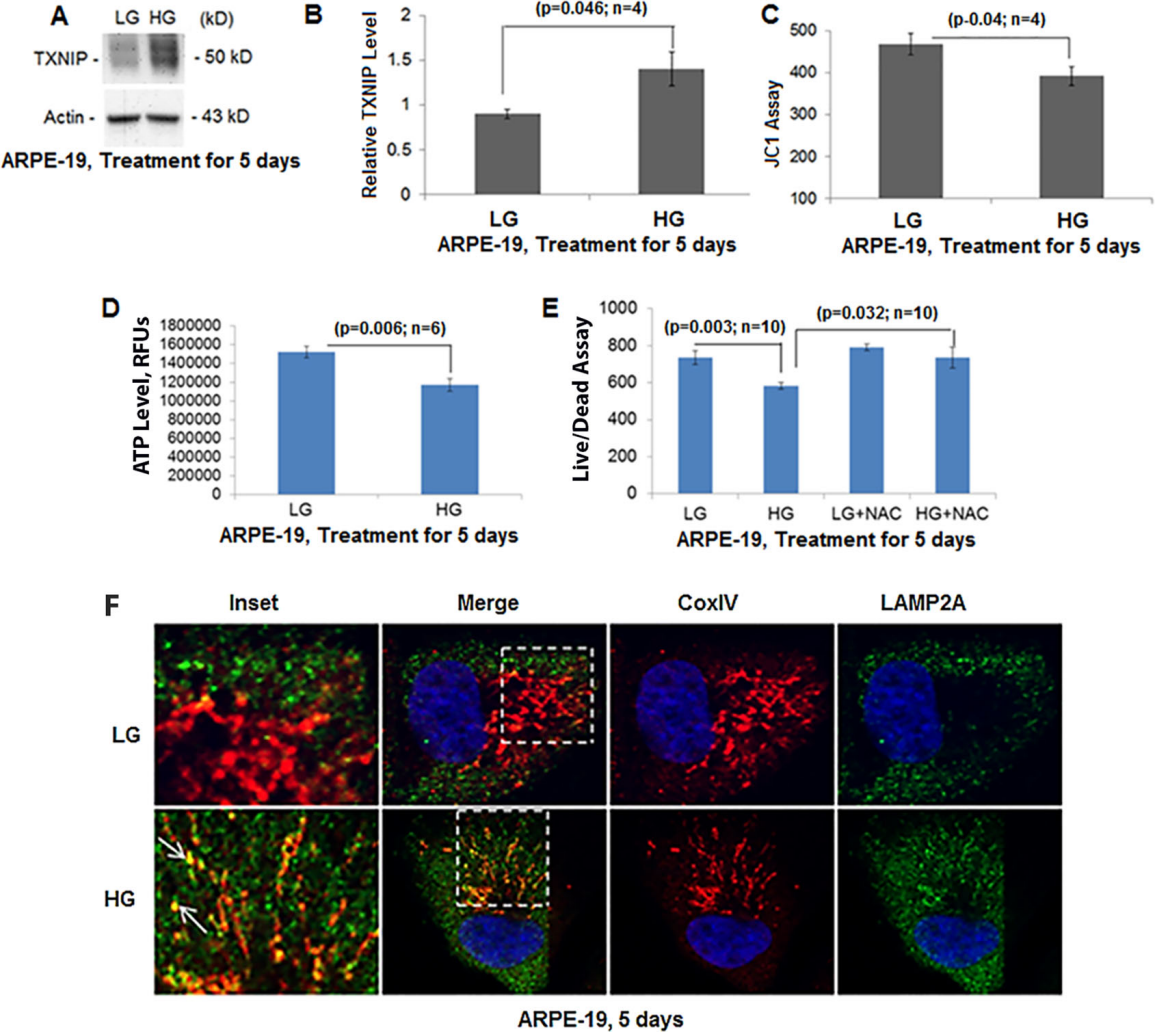
**High glucose induces TXNIP expression and mitochondrial dysfunction in ARPE-19 cells.** (A) Total cell extracts were prepared in RIPA buffer and TXNIP levels were detected on western blots. (B) On densitometric analysis, HG for 5 days significantly (*P*<0.05; *n*=4) increases TXNIP levels in ARPE-19 cells compared to LG. (C) HG also causes a reduction in mitochondrial membrane potential as detected by JC1 assay. (D) Correspondingly, ATP levels are reduced significantly (*P*=0.0006; *n*=6) by HG. (E) Cell viability is reduced under HG compared to LG, which is prevented by the antioxidant NAC (5 mM). (F) In agreement, mitochondrial ETC complex IV protein CoxIV co-localizes the lysosomal membrane protein LAMP2A (inset, arrows) indicating mitophagic flux to lysosomes. Fixed cell images were captured in a Zeiss confocal microscope at ×630 magnification. A representative image of *n*=3 is shown.

Treatment of ARPE-19 for 5 days with HG leads to significant increases in TXNIP expression (at both the mRNA and protein levels) when compared to LG conditions (Fig. 1A,B and Fig. S1). TXNIP induction under HG is also associated with mitochondrial membrane depolarization, as shown by a reduction in JC1 in Fig. 1C and decreases in ATP levels in Fig. 1D. Furthermore, cell viability is reduced under HG but this is rescued by the antioxidant N-acetylcysteine (NAC) (Fig. 1E), suggesting that oxidative stress is involved in this process. Furthermore, the TXNIP level is also increased in the mitochondrion while mitochondrial Trx2 is reduced (Fig. S2B). Damaged mitochondria are removed by an autophagic process via lysosomal degradation. Indeed, the levels of the mitophagy/autophagy markers LC3BII and p62/SQSTM1 (Sequestosome 1) are significantly reduced under HG (Fig. S2C), suggesting lysosomal degradation. We further show increases in mitochondria targeted to lysosomes under HG by co-localization of the mitochondrial protein CoxIV with the lysosomal membrane protein LAMP2A in immunostaining (Fig. 1F), further supporting a mitophagic flux under HG in ARPE-19 cells. We also observed co-localization of mitochondrial antioxidant Trx2 and LAMP2A under HG (Fig. S3), further supporting that HG induces mitophagic flux in ARPE-19.

### Mitochondrial fragmentation and mitophagic flux cause lysosomal enlargement in RPE cells

To analyze whether mitophagic flux leads to lysosomal enlargement, we transiently transfected ARPE-19 cells with a plasmid containing Ad-CMV-LAMP1-mCherry. After treatment with HG for 5 days, the lysosomal sizes significantly increased (two- to threefold) compared to LG treatment (Fig. S4A,B). In addition, primary human HRPE cells were also co-transfected with lysosomal LAMP1-mCherry for 3 days and mitochondria-targeted GFP (mt-GFP) for 24 h during 5 days of HG treatment (Fig. S4C). Here again, the lysosomal sizes are significantly increased in HRPE cells under HG (Fig. S4D,F,G) compared with LG. Mitochondria mt-GFP filaments in HRPE cells are fragmented under HG compared with LG (Fig. S4E,H). Mito-fission is a pre-requisite for mitochondrial autophagosome formation.

**Fig. 2.**
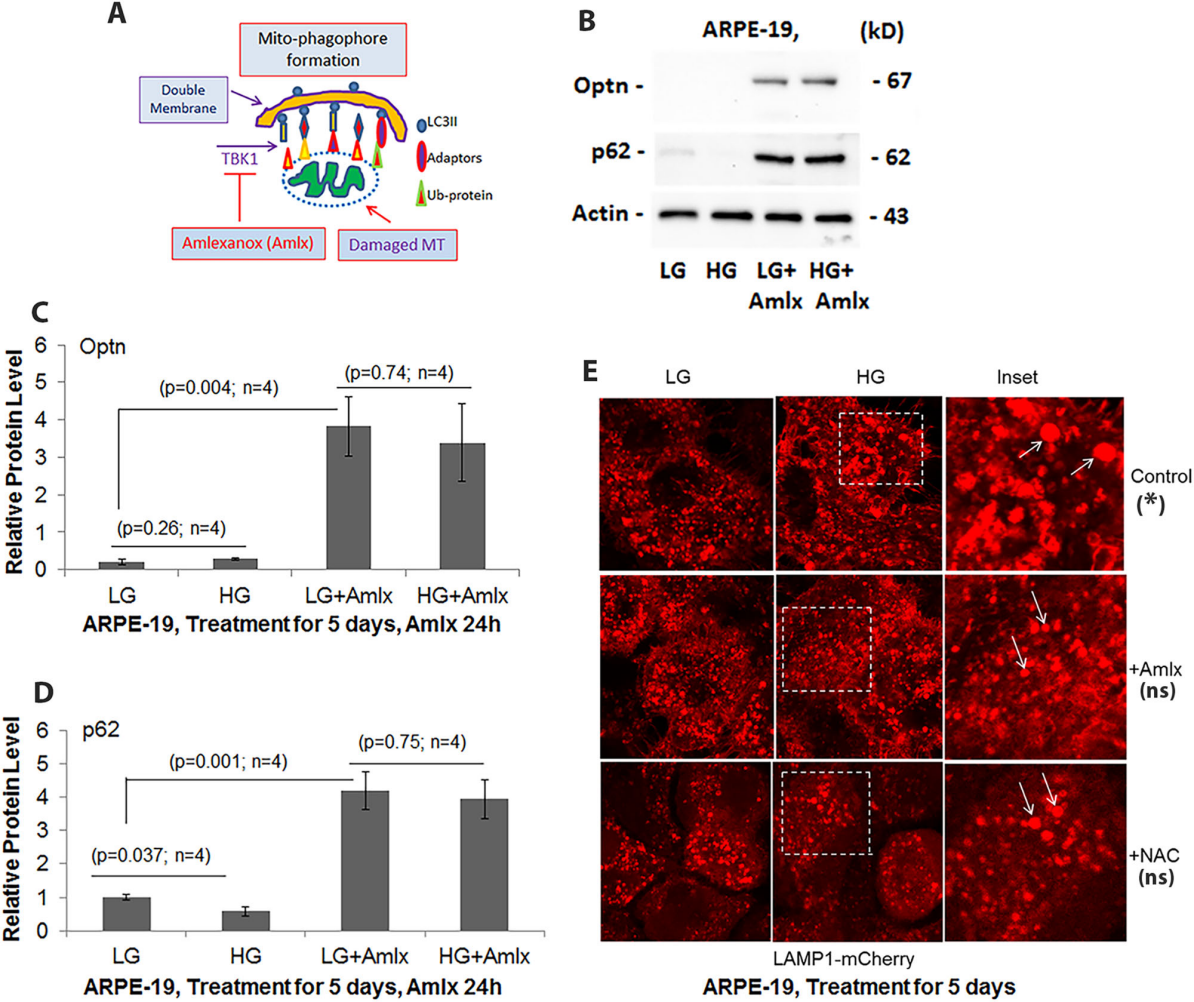
**TBK1 inhibitor Amlx prevents mitophagic flux and lysosome enlargement in ARPE-19 cells.** (A) TBK1 is known to phosphorylate mitophagy adaptors such as Optn and p62/SQSTRM1 and increases their mitophagic activities. (B) Inhibition of TBK1 by Amlx (1 µM) reduces HG-induced mitophagic flux as indicated by increased levels of Optn and p62/SQSTRM1 (p62) in ARPE-19 cells. HG treatment was for 5 days while Amlx and was present for the final 24 h before harvesting the cells. A blot image of *n*=4 is shown here. (C) Denitometric data analysis of Optn and (D) p62/SQSTM1. (E) Ad-CMV-LAMP1-mCherry was transduced transiently in ARPE-19 cells for 3 days. Lysosomes containing the LAMP1-mCherry, are seen enlarged under HG (upper panels, arrows) compared to LG. Amlx (1 µM) and NAC (5 mM), when present for 24 h, also reduce lysosomal size under HG in ARPE-19 (middle and lower panels, respectively, arrows). Fixed cell images were captured in a Zeiss confocal microscope at ×630 magnification. A representative image of *n*=3 is shown. * indicates significant lysosomal size enlargement by HG when compared with LG (*P*<0.05; *n*=15) while ns represents no significant change in lysosome sizes between HG and LG.

To further ascertain whether the mitophagic flux is responsible for the enlarged lysosomes under HG, we treated ARPE-19 cells with Amlexanox (Amlx), an inhibitor of TBK1 (TANK Binding Kinase 1). TBK1 enhances mitophagic flux via phosphorylation of mitophagy adaptors, such as Optineurin (Optn) and p62/SQSTM1 (Fig. 2A). Phosphorylation of these adaptors by TBK1 increases their ubiquitin-binding activities and LC3BII interactions and lysosomal degradation. Therefore, Amlx treatment will block mitophagic flux by inhibitng TBK1. We observed that under both LG and HG, the levels of Optn and p62/SQSTM1 are low (Fig. 2B), suggesting an active basal mitophagic flux in ARPE-19. Amlx treatment, however, increases both Optn (Fig. 2C) and p62/SQSTM1 (Fig. 2D) levels, indicating an inhibition of mitophagic flux and degradation in lysosomes. Amlx also prevents HG-induced lysosomal enlargement, which is more or less comparable to that seen under LG conditions (Fig. 2E, upper versus middle panels), suggesting that mitophagic flux leads to lysosomal enlargement. Furthermore, the antioxidant NAC reduces HG-mediated increases in lysosome size (Fig. 2E, lower panels). In addition to Amlx, Bafilomcyin A (Baf-A), which prevents the lysosome-autophagosome fusion, also increases optn and p62/SQSTM1 (Fig. S5).

### Mt-Keima as a fluorophore probe to measure mitophagic flux by live-cell imaging

Although mt-GFP shows that mitochondria are fragmented, mt-GFP appears to be quenched in lysosomes under an acidic environment; therefore, we could not adequately measure lysosome targeting (Fig. S3C). Therefore, to confirm the mitophagic flux to lysosomes under HG, we further developed an Ad-CMV promoter-mt-Keima with the mitochondrial target sequence of COXVIII. As mentioned before, mt-Keima is a coral protein that emits green light (Ex485 nm) in the alkaline environment inside mitochondria and emits red light in acidic lysosomes (Ex561 nm) via the mitophagic flux. However, both excitations give a single emission peak at 620 nm, and therefore the same mt-Keima can be used to monitor mitophagic flux to lysosomes.

**Fig. 3.**
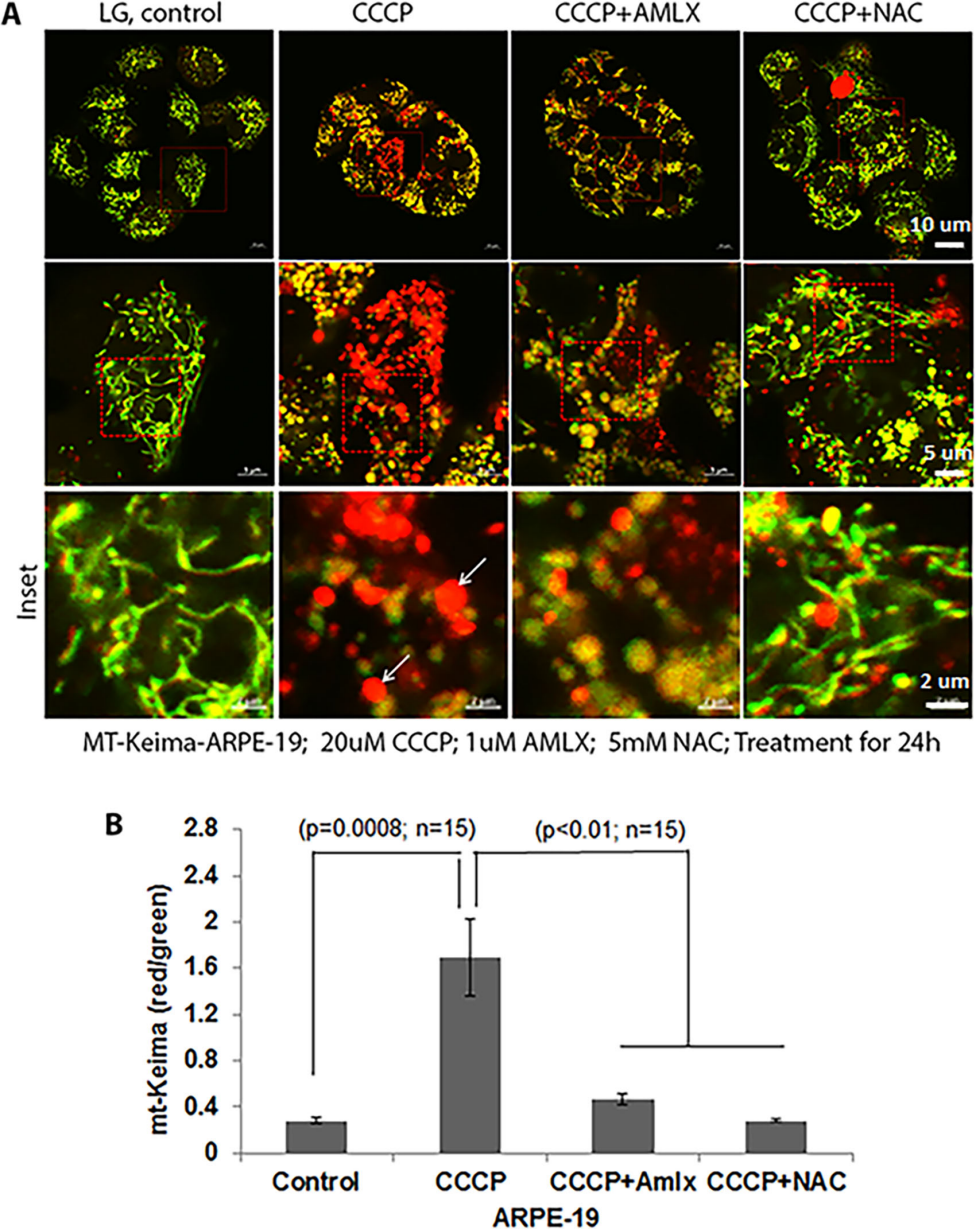
**Mt-Keima as a mitophagy probe.** ARPE-19 cells were transduced with Ad-CMV-mt-Keima for 3 days under LG conditions. CCCP, (20 µM) a mitochondrial inner membrane ionophore, was added for 24 h. When present, Amlx (1 µM) and NAC (5 mM) were together with CCCP for 24 h. (A) In ARPE-19 cells, CCCP induces mitophagic flux (enlarged red mt-Keima) compared to in LG. Most mt-Keima in LG remain green filaments, indicating a mitochondrial network (inset). Arrows show red mt-Keima in lysosomes. Both Amlx and NAC reduce red mt-Keima indicating a blockade of mitophagic flux. Live images were captured in a Zeiss confocal microscope at ×630 magnification. A representative image of *n*=3 is shown. (B) Quantitation of mt-Keima red/green shows significant increases in mitophagic flux by CCCP significantly, which is prevented in the presence of Amlx and NAC.

To confirm mt-Keima distribution in mitochondria and to lysosomes after mitophagic flux (green versus red mt-Keima, respectively), we first used Carbonyl Cyanide 3-ChloroPhenyl-hydrazone (CCCP), a mitochondrial inner membrane ionophore, to depolarize the mitochondrial membrane and activate mitophagy. For this, we transiently transduced the Ad-CMV-mt-Keima plasmid for 3 days and then treated the cells with 20 µM CCCP for 24 h under LG conditions. As shown in Fig. 3A, without CCCP, mt-Keima emits predominantly green fluorescence, suggesting mitochondrial localization, as captured by confocal live-cell microscopy. However, a majority of the mt-Keima emits red fluorescence after treatment with CCCP, indicating mitophagic flux to lysosomes (Fig. 3A, CCCP inset). In the presence of both CCCP and Amlx or NAC, however, mt-Keima emits green fluorescence, confirming the blockade of the mitophagic flux. Quantitation of the mt-Keima red/green ratio (lysosome/mitochondria) shows a significant mitophagic flux induced by CCCP in ARPE-19 and inhibition induced by the presence of Amlx and/or NAC (Fig. 3B).

**Fig. 4.**
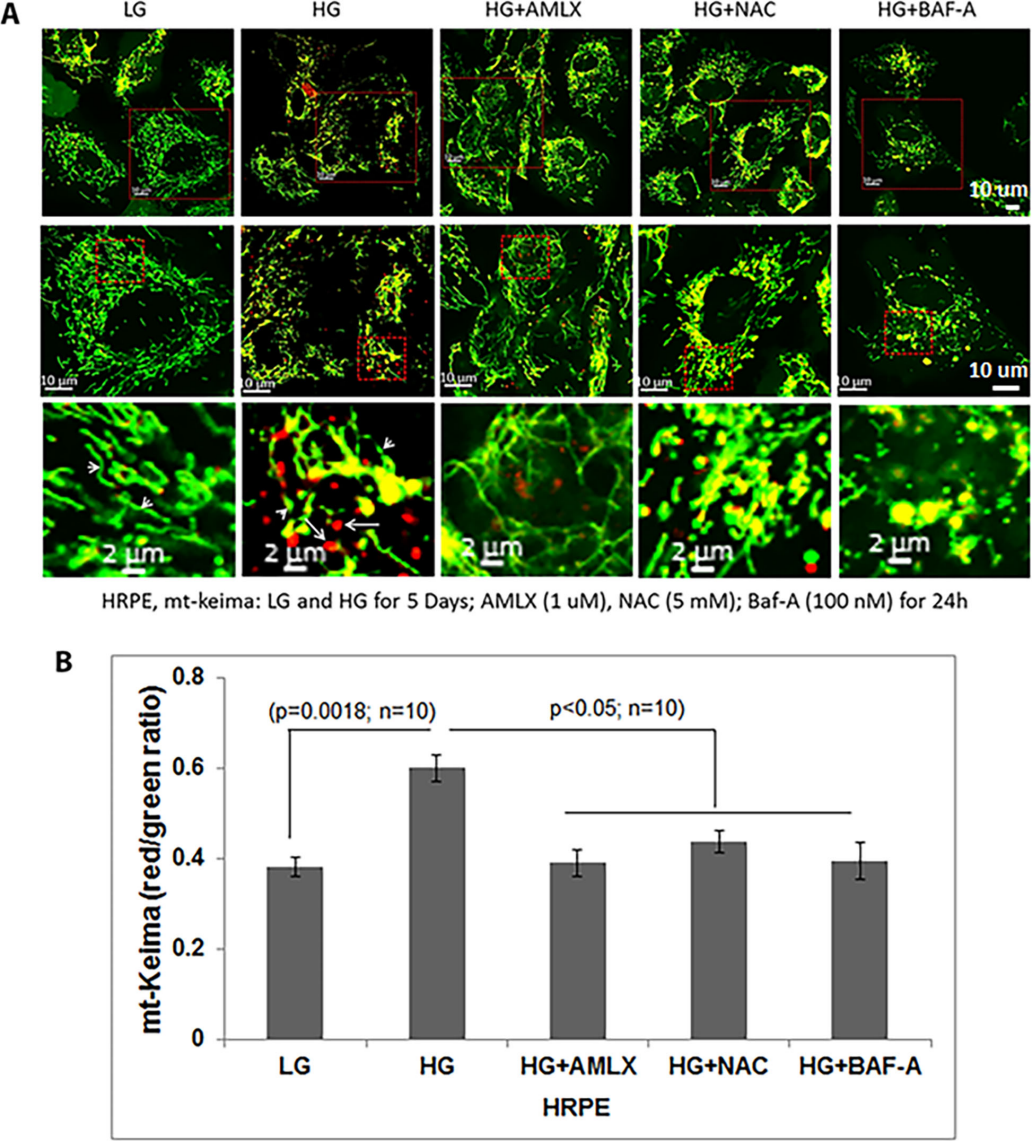
**High glucose induces mitophagy in human** **primary HRPE cells.** (A) HRPE cells were treated with LG or HG for 5 days in six-well plates containing microscopic slide coverslips. Ad-CMV-mt-Keima was transfected for 3 days. Under LG, mt-Keima is seen as green filaments in mitochondria (lower panel, arrowheads) while under HG, mt-Keima is seen as red in lysosomes and mitochondria are fragmented (lower panel, arrows). Several drugs such as Amlx, NAC and Baf-A prevent the mitophagic flux to lysosomes in HRPE cells as there is predominantly green mt-Keima and less red puncta in the presence of these chemicals. Images were captured in a Zeiss confocal microscope at ×630 magnification. A representative image of *n*=3 is shown. (B) Quantitation of the mt-Keima red/green in HRPE cells after different treatments seen in A is shown.

Further, when mt-Keima was transduced into primary HRPE cells, mitochondria are seen as elongated networks of green mt-Keima (arrowhead) and smaller red lysosomes under LG conditions, indicating basal mitophagy (Fig. 4A). However, HG treatment causes mitochondrial fission (fragmented green mt-Keima) and enlarged lysosomes (red mt-Keima, inset, arrows). In addition, Amlx, NAC and bafliomycin A (Baf-A), which disturb the lysosomal membrane and inhibit autophagosome-lysosome fusion, prevent mt-Keima flux to lysosomes in HRPE cells. This is revealed by the enlarged red mt-Keima seen under HG conditions but not in the presence of these inhibitors (Fig. 4A,B). In support of mitophagic flux in HRPE cells under HG, we also show that Baf-A, which prevents lysosome-autophagosome fusion cargo degradation, increases LC3B, Optn and p62 levels both in LG and HG, indicating a blockade of mitophagic flux (Fig. S6). Therefore, mt-Keima represents an excellent probe to measure multiple properties of mitochondrial fragmentation, mitophagic flux and lysosome enlargement under cellular stresses in living cells.

### TXNIP knockdown prevents mitochondrial damage and mitophagic flux in RPE cells

**Fig. 5.**
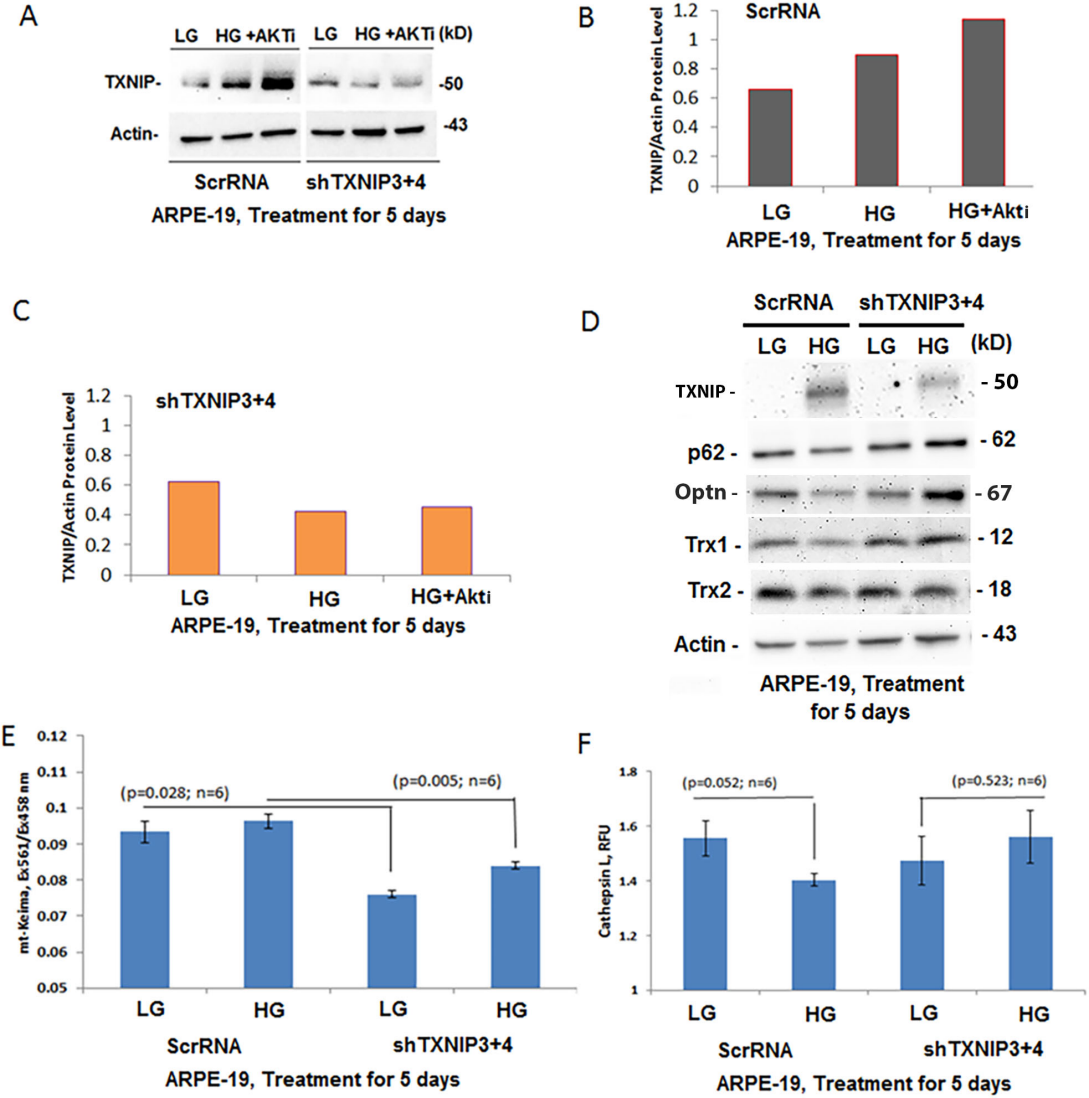
**TXNIP knockdown by shRNA in ARPE-19 cells.** Various shRNAs targeted to TXNIP (shTXNIP 1, 2, 3 and 4 in pcDNA3.1 plasmid) and their combinations were tested for TXNIP knockdown in ARPE-19 cells after stable cell selection. A combination of shTXNIP3+4 was found most effective and we use it to knock down TXNIP while a scramble shRNA (ScrRNA) was used as a control. Stable cell lines were selected using G417. (A,B) HG increases TXNIP level in ScrRNA control ARPE-19 cells which further increased in the presence of an AKT inhibitor (0.5 µM AKT inhibitor VI, AKTi). Akt is known to phosphorylate and degrade TXNIP protein. (A,C) Conversely, in shTXNIP3+4 ARPE-19 cells, HG or HG+AKTi failed to enhanced TXNIP levels, suggesting a knockdown of TXNIP (*n*=2). (D) HG also decreases p62/SQSTM1 (p62) and redox proteins Trx1 and Trx2 in scrRNA control ARPE-19 cells. These changes in protein levels are reversed in shTXNIP3+4 ARPE-19 cells, suggesting a role for TXNIP in redox imbalance and mitophagic flux. A representative of *n*=3 is shown. Densitometric analysis is shown in Fig. S4. (E) In fact, mt-Keima (red/green) ratio, an indicator of mitophagic flux, is also reduced in shTXNIP3+4 cells when compared to scrRNA ARPE-19 cells as measured by a fluorescent microplate reader using Ex561 nm (red) and Ex458 nm (green) while keeping emission at 620 nm for both excitations. (F) HG also reduces lysosomal enzyme cathepsin L activity in ScrRNA ARPE-19 cells while it is prevented in shTXNIP3+4 cells.

We next investigated the role of TXNIP in mitophagic flux, lysosome enlargement and destabilization after TXNIP knockdown by shRNA in ARPE-19 cells. In control cells, which expressed scramble RNA (scrRNA), HG exposure increases the TXNIP protein level and more so in the presence of an Akt inhibitor and HG together (Fig. 5A,B). Akt is known to phosphorylate and degrade TXNIP. After knocking down TXNIP with a mixture of TXNIP shRNA #3 and #4 (shTXNIP3+4) in APRE-19 cells, a ∼70% reduction in the TXNIP level was seen (Fig. 5B,C). The effectiveness of TXNIP knockdown is supported by the inability of HG or HG+Akt inhibitor to increase TXNIP levels in shTXNIP3+4-transfected ARPE-19 cells. Furthermore, TXNIP knockdown also restores redox proteins, such as Trx1 and Trx2, as well as mitophagy adaptors Optn and p62/SQSTM1 under HG conditions (Fig. 5D and their densitometric quantitation in Fig. S7). In addition, the mt-Keima red/green ratio is increased by HG in scrRNA ARPE-19 cells, but not in shTXNIP3+4 cells, when measured by a fluorescent microplate reader (Fig. 5E). Furthermore, the activity of the lysosomal enzyme cathepsin L is decreased by HG in scrRNA cells, but not in shTXNIP3+4 cells (Fig. 5F), suggesting that excess mitophagic flux may cause lysosomal destabilization.

**Fig. 6.**
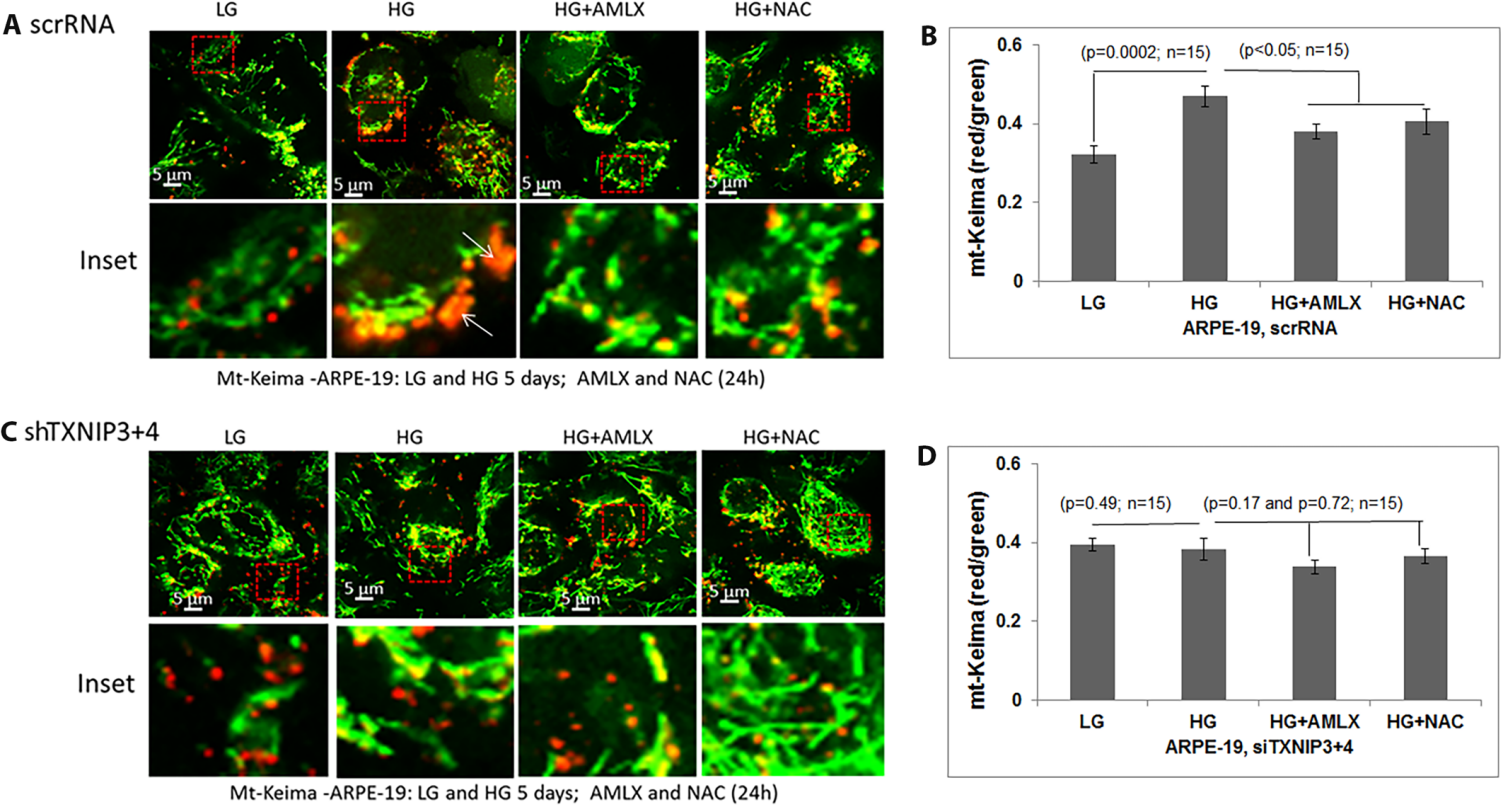
**TXNIP knockdown prevents mitophagic flux in ARPE-19 cells.** (A) In ScrRNA control ARPE-19 cells, HG (5 days) induces mitophagic flux (enlarged red mt-Keima) as opposed to in LG. Amlx (1 µM) and NAC (5 mM), which were added 24 h before taking the images, prevents red mt-Keima formation, indicating an inhibition of mitophagic flux. Ad-CMV-mt-Keima was transduced transiently for 3 days. Arrows show red mt-Keima in lysosomes. (B) Quantitation of the red/green mt-Keima shows significant increase in mitophagic flux, which is reduced by Amlx and NAC. (C) Effect of HG on mitophagic flux is absent in shTXNIP3+4 cells, indicating a role for TXNIP in HG-induced mitophagic flux in ARPE-19 cells. Furthermore, Amlx and NAC effects on red mt-Keima are absent in shTXNIP3+4 ARPE-19 under HG. Live cell images were captured in a Zeiss confocal microscope at ×630 magnification. A representative image of *n*=3 is shown. (D) mt-Keima red/green quantitation is shown. No significant change in mt-Keima red/green ratio is seen in shTXNIP3+4.

After Ad-CMV-mt-Keima transduction in scrRNA ARPE-19 cells, we further observed enlarged red mt-Keima puncta after HG treatment (Fig. 6A,B), indicating its accumulation in lysosomes; this, however, was not seen in shTXNIP3+4 APRE-19 cells (Fig. 6C,D). Amlx and NAC reduce red mt-Keima in scrRNA control APRE-19 cells (Fig. 6A,B), but no additional effects are seen on shTXNIP3+4 cells (Fig. 6C,D). Interestingly, hydrogen peroxide (H_2_O_2_)-induced mitophagic flux is also prevented by TXNIP knockdown in shTXNIP3+4 ARPE-19 cells, but not in scrRNA control cells (Fig. S8A,B versus C,D), further reinforcing the idea that TXNIP has a role in redox regulation and oxidative stress-induced mitophagic flux in RPE cells.

### TXNIP knockdown prevents HG-induced lysosome enlargement and destabilization in RPE cells

**Fig. 7.**
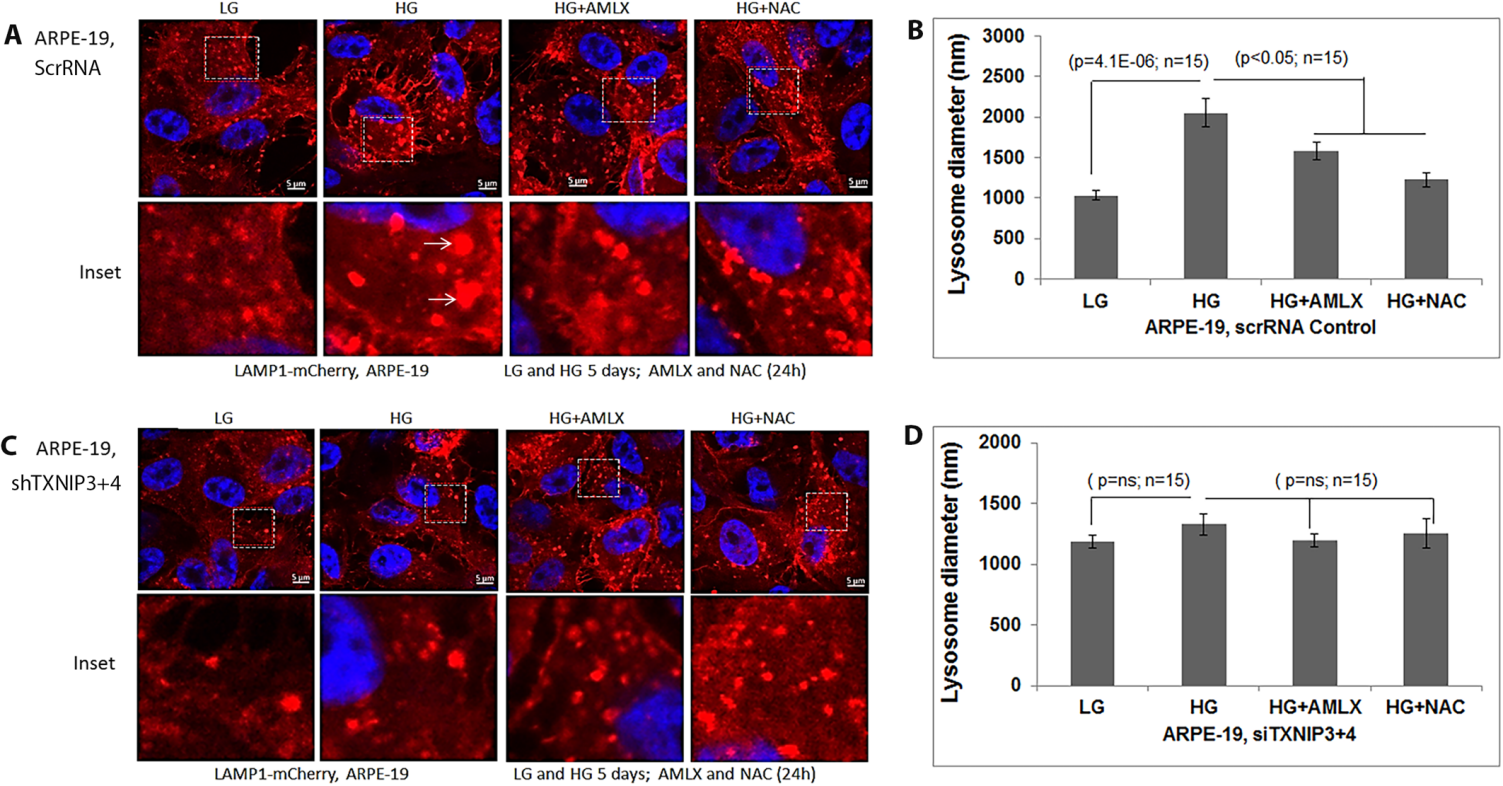
**TXNIP knockdown prevents lysosome enlargement in ARPE-19 cells.** (A) In ScrRNA control ARPE-19 cells, HG (5 days) induces lysosome enlargement (LAMP1-mCherry) compared to LG, which is reduced by Amlx (1 µM) and NAC (5 mM). Ad-CMV-LAMP1-mCherry was transduced for 3 days while Amlx and NAC were added 24 h before taking the images. Arrows show enlarged lysosomes under HG. (B) Quantitation of lysosome sizes in scrRNA ARPE-19 cells with HG in the absence or presence of Amlx and NAC. Significant lysosome size increase is seen with HG but not in the presence of Amlx or NAC. (C,D) The effect of HG on lysosome enlargement is absent in shTXNIP3+4 ARPE-19 cells, indicating a role for TXNIP in HG-induced lysosome enlargement in ARPE-19 cells. A representative image of *n*=3 is shown.

We also observed HG induction of enlarged lysosomes in LAMP1-mCherry-expressing scrRNA control ARPE-19 cells when compared to LG conditions (Fig. 7A,B). Amlx and NAC prevented the HG-induced lysosomal enlargement more or less comparably to the level under LG conditions (Fig. 7B). Conversely, in stable TXNIP knockdown ARPE-19 (shTXNIP3+4) cells, the lysosome size remains similar in both the LG and HG conditions (Fig. 7C,D).

Lysosome destabilization under HG and mitophagic flux may also lead to inflammasome activation. In agreement, we observed that HG increases caspase-1 activity in ARPE-19 cells, which is prevented by NAC (Fig. S9A). While caspase-1 mRNA expression is not significantly changed by HG (Fig. S9B), that of NLRP3 and pro-IL-1β are enhanced significantly (Fig. 6C,D), suggesting inflammasome activation in ARPE-19. In support of enhanced caspase-1 activity upon lysosomal membrane destabilization/permeabilization (LMP), we also observed that L-Leucyl-L-Leucine methyl ester (LLME), which induces LMP, significantly increased caspase-1 activity and decreased cathepsin L activity (Fig. S10A) in ARPE-19. Amlx itself has no effect on either caspase-1 or cathepsin L activity (Fig. S10B). Furthermore, LLMe reduced cellular ATP levels, whereas Amlx had no effect. Altogether, the results indicate that TXNIP is critically involved in HG-induced mitochondrial dysfunction, mitophagic flux and lysosome enlargement/destabilization and caspase-1 activation in RPE cells. Amlx may inhibit some of the deleterious effects of HG and TXNIP on RPE.

## DISCUSSION

**Fig. 8.**
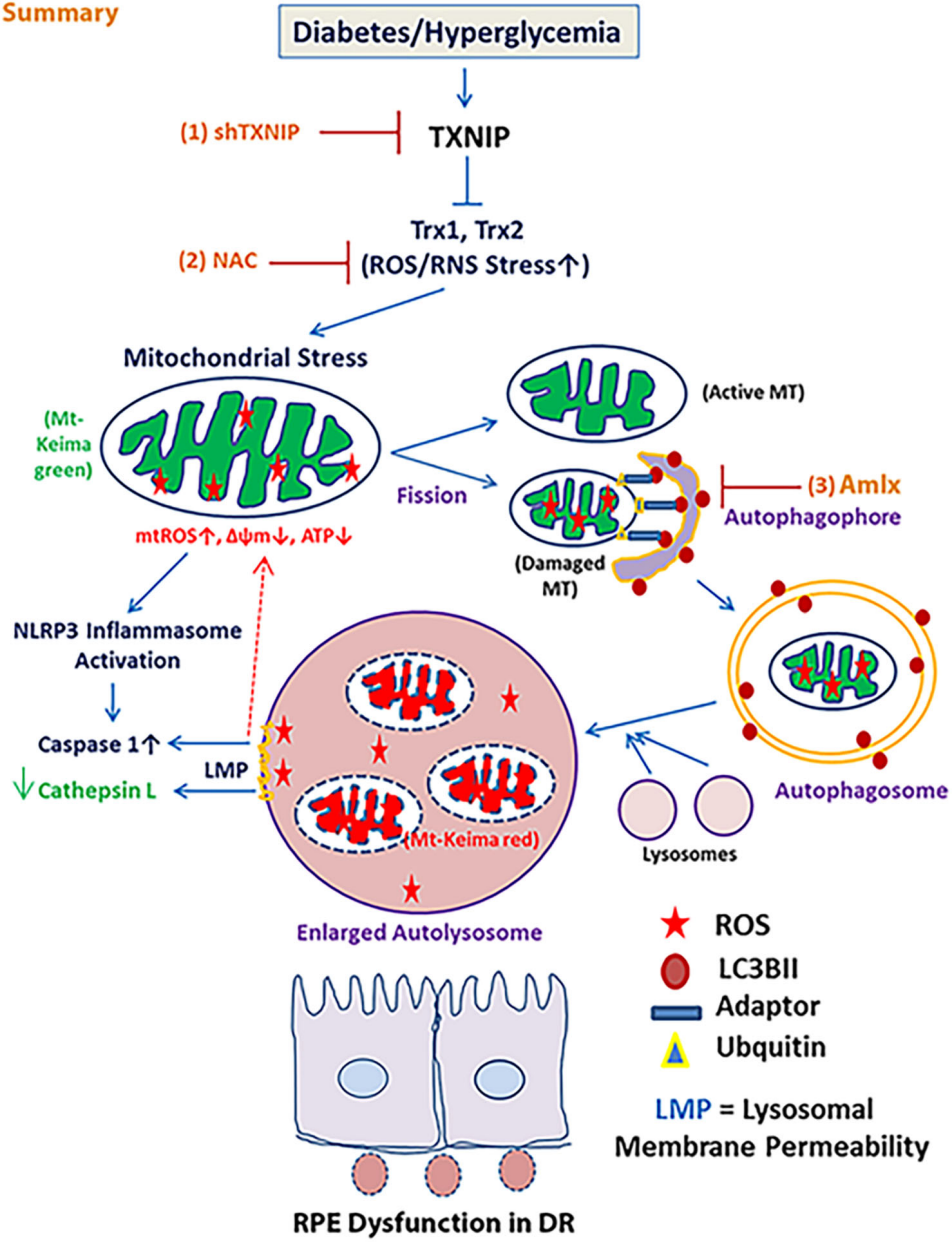
**A potential role for TXNIP in HG-mediated RPE dysfunction.** Diabetes and hyperglycemia upregulates TXNIP and causes cellular ROS/RNS stress, mitochondrial dysfunction, mitophagic flux, lysosome enlargement/destabilization, inflammation and RPE dysfunction. Released lysosomal enzymes may degrade mitochondrial membrane proteins and a vicious cycle of mitochondria–lysosome pathway dysfunction may be established. TXNIP knockdown or mitochondrial targeted antioxidants may prevent several deleterious effects of high glucose and TXNIP on RPE in DR.

In this manuscript, we demonstrate that (i) HG-induced TXNIP upregulation in human RPE cells is associated with mitochondrial dysfunction and fragmentation, (ii) dysfunctional (depolarized) mitochondria are removed by mitophagy targeted to lysosomes, (iii) mitophagic flux and overloading of damaged mitochondria to lysosomes cause lysosomal enlargement and inactivation of lysosomal enzymes (such as cathepsin L), (iv) lysosomal destabilization may lead to NLRP3 inflammasome activation (as indicated by caspase-1 activation and increased expression of NLRP3 and IL-1β mRNA), and (v) TXNIP knockdown by shRNA prevents mitochondrial fragmentation, mitophagy and lysosome enlargement. Furthermore, the antioxidant NAC and Amlx ­– a TBK1 kinase inhibitor – block mitophagic flux and lysosome enlargement, suggesting that oxidative stress and the phosphorylation of the mitophagic adaptors Optn and p62/SQSTM1 by TBK1 may play a role in mitophagic flux in RPE cells under diabetic conditions. A summary diagram is shown in Fig. 8.

Accumulation of fragmented and damaged mitochondria generate ROS while producing little ATP (bioenergetics failure). Mt-ROS and mitochondrial components are recognized by cytosolic pattern recognition receptors, such as cytosolic NLRP3 inflammasomes and endosomal/lysosomal TLR4, TLR3/7, and TLR9 (Hughes and O'Neill, 2018; Singh et al., 2017). The result is the activation of caspase-1 and pro-inflammatory cytokines, such as IL-1β and IL-18 and inflammation. Therefore, the removal of the damaged mitochondria is critically important for mitochondrial health and cell survival. However, when the insult to cells continues, as seen in chronic hyperglycemia, mitochondrial damage occurs, leading to fragmentation (fission) and mitophagic flux to lysosomes. Overloading of damaged mitochondria to the lysosome leads to its enlargement, which probably involves lysosome and autophagosome as well as multiple late endosome-lysosome fusion (Carroll and Dunlop, 2017; Henry et al., 2015). This event leads to lysosomal destabilization (alkalization), causing the inactivation of acid hydrolases, including cysteine proteases (cathepsin B, D, K, L and others). Accumulation of mitochondrial membrane lipids, aggregated proteins and iron-sulfur complexes leads to lipofuscin formation and also to lysosomal oxidative stress via the Fenton reaction, which generates highly reactive hydroxyl ions (König et al., 2017; Terman and Kurz, 2013). These damaging reactive hydroxyl ions disturb lysosomal membranes and induce LMP. Lysosomal cathepsins leak out into the cytosol and can further degrade mitochondrial outer membrane proteins (Zhou et al., 2017). These events thus generate a vicious cycle of mitochondrial damage–mitophagic flux–lysosome damage and ultimately cell death, either by apoptosis or inflammatory pyroptosis. At later stages, LMP may also lead to a failure of autophagosome–lysosome fusion, resulting in accumulation of damaged mitochondria and autophagosomes, which further causes cytosolic crowding, loss of sanctity, ROS generation, and autophagic cell death.

HG levels increase TXNIP expression in both mitochondria and the cytosol and decrease the expression of redox proteins, such as cytosolic Trx1 and mitochondrial Trx2, suggesting that hyperglycemia induces RPE redox stress. We further observe that HG decreases antioxidant Cu/Zn superoxide dismutase 1 (SOD1) and tight junction protein zonula occludens 1 (ZO1) expression in RPE cells (data not shown). Such events may cause oBRB leakage, the accumulation of blood components into subretinal spaces, and photoreceptor degeneration (Desjardins et al., 2016). Indeed, photoreceptors have been shown to generate large amounts of ROS in early DR (Du et al., 2013), which may influence microvascular dysfunction (Fu et al., 2018). Therefore, it is tempting to speculate that RPE dysfunction in early DR may be one mechanism by which photoreceptor injury, Müller cell activation and microvascular damage occur (Tonade et al., 2017). Such events may begin with an induction of TXNIP, oxidative stress, mitochondrial damage, excess fission, mitophagic flux and lysosome enlargement/destabilization (Devi et al., 2017; Singh et al., 2017). These events may reduce POS phagocytosis and retinol recycling by RPE cells. Interestingly, in most neurodegenerative diseases – such as ALS-amyotrophic lateral sclerosis, Parkinson's and Alzheimer's – SOD1 dysfunction, mitochondrial fragmentation and bioenergetics deficiencies occur (Trist et al., 2017; Van Damme et al., 2017); similar events may occur in diabetic retinal neurodegeneration.

Mitophagy is a complex process in which the depolarized mitochondria accumulate PINK1 at its outer membrane (Deas et al., 2011; Mouton-Liger et al., 2018). PINK1 is normally transported to the mitochondrial inner membrane and degraded by PARL protease (Deas et al., 2011); however, damaged mitochondria fail to translocate PINK1 and phosphorylated outer membrane proteins and ubiquitin. This then attracts the translocation of cytosolic Parkin to damaged mitochondria (Durcan and Fon, 2015). Parkin ubiquitinates Pink1-phosphorylated membrane proteins, including VDAC1 and Mfn2, and they are tagged for engulfment by a double membrane phagophore containing LC3BII (Durcan and Fon, 2015). Mitophagy adaptors, such as Optn and p62/SQSTM1, recognize both LC3BII and ubiquitinated mitochondrial membrane proteins to facilitate autophagosome formation (Heo et al., 2015). The binding of Optn and p62/SQSTM1 to ubiquitinated proteins and LC3BII are enhanced by phosphorylation of these adaptors by TBK1 (Bodur et al., 2018; Oakes et al., 2017). TBK1 is a protein kinase of the non-conventional IKKε/TBK1 pathway, which is also involved in anti-viral activities and inflammation by targeting the transcription factors NF-κB and IRF3/7 (interferon responsive factors) and inducing pro-inflammatory cytokines, such as pro-IL-1β and interferon-β, respectively (Bodur et al., 2018). The fact that Amlx, an inhibitor of TBK1 (Minegishi et al., 2016; Oral et al., 2017) that blocks mitophagic flux by reducing Optn and p62/SQSTM1 activities, reduces lysosome size suggests that Amlx may be a potential drug to regulate excess mitophagic flux and maintain lysosomal function under hyperglycemic redox stress.

Some studies have shown that HG and TXNIP inhibit mitophagy on the basis of increased p62/SQSTM1 levels in renal proximal tubules (Huang et al., 2016). P62/SQSTM1 is also involved in Nrf2 activation via Keap1 degradation. Nrf2 itself is able to induce p62/SQSTM1 expression. Mitophagy is known to induce both TFEB and Nrf2 to regulate lysosomal and autophagy/mitophagy gene expressions (Ivankovic et al., 2016; Jung et al., 2016). Therefore, increases in p62/SQSMT1 under certain stresses may be a stress response rather than an inhibition of mitophagy. Furthermore, different cell types may have different mitophagic responses under similar environments. Therefore, mitophagic flux measurements based on LC3B and/or p62 level alone may not reflect a true mitophagic flux. Mitophagosome targeting to lysosomes as well as detection of lysosomal morphology and lysosomal enzyme activity need to be incorporated into such mitophagy studies, as seen in this study. We observed that the antioxidant NAC prevents HG-induced mitochondrial fragmentation, mitophagic flux and lysosome enlargement, further suggesting that mito-targeted antioxidants could be used to maintain RPE function as well. Unlike the neuroretina, RPE is accessible to systemic drug treatment via fenestrated choriocapillaris. In addition, gene therapy for TXNIP knockdown may also be an attractive approach to reduce oxidative stress and mitochondrial dysfunction not only in retinal RPE dysfunction, but also in other retinal cells in early DR. This is because TXNIP is induced by HG in most retinal cells, including capillary endothelial cells, pericytes, Müller cells and microglia as well as RPE as shown here (Devi et al., 2012, 2013, 2017; Kowluru and Mishra, 2018; Perrone et al., 2009, 2010; Singh et al., 2013, 2017).

The mechanisms as to how mitochondrial damage signals induce LC3BII double-membrane formation and lysosome targeting are not fully understood yet. In this regard, TXNIP has been shown to interact with REDD1 and induces oxidative inhibition of ATG4B, which increases LCB3II phagophore formation (Qiao et al., 2015). Furthermore, we show here that HG increases TXNIP expression and reduces the expression of the redox proteins Trx1, Trx2 and SOD1. SOD1 is present in both the cytosol and mitochondrial intermembrane space. These events further cause cellular oxidative stress, protein oxidation and inactivation of ATG4B, and subsequently increase LC3BII available for autophagophore formation. Furthermore, under HG, TXNIP induction and oxidative stress may inactivate Akt (Ahmad et al., 2014), which further increases TXNIP expression in a feed-forward manner. Akt has been shown to phosphorylate TXNIP at serine 308 and enhances degradation (Waldhart et al., 2017). Further, Akt also targets mTORC1 and activates its activity to suppress autophagy. Therefore, oxidative inhibition of Akt may activate TXNIP by suppressing mTORC1 (Kaadige et al., 2015). In this regard, TXNIP silencing itself prevents hydrogen peroxide (H_2_O_2_)-induced mitophagic flux in APRE-19 cells (Fig. S8). This result suggests that TXNIP is involved in both HG- and oxidative stress-induced mitophagy, further supporting the notion that TXNIP knockdown is a potential mechanism to maintain the homeostasis of the mitochondria–lysosome axis.

Mt-Keima is an excellent tool to study mitophagic flux under normal and disease conditions. We used mt-Keima in this study as a mito-probe to directly demonstrate the role of TXNIP in HG-induced mitophagic flux to lysosomes in live RPE cells by using confocal cell imaging. Our mt-Keima results demonstrate mitochondrial fragmentation, mitophagic flux and lysosomal enlargement, both in ARPE-19 and primary HRPE cells. Recent studies have also shown that mt-Keima can be used to study mitophagic flux in animal models (Singh et al., 2017; Sun et al., 2017; Zhou et al., 2011). Mt-Kiema is resistant to acid hydrolases in lysosomes, and therefore red mt-Keima accumulates in lysosomes longer, allowing the researcher time to detect it. Nonetheless, using mt-Keima in live tissue without fixation could be challenging for certain tissues, such as the neuroretina, which is fragile and forms only a tiny layer of the eye. Furthermore, processing and measuring live cells for multiple samples in a time-dependent manner is inconvenient for many experimental approaches. If one has to fix the cells, the lysosomal acidic environment is lost during fixation and then mt-Keima will emit green fluorescence in both the mitochondria and lysosomes.

For the above reasons, we further used an Ad-CMV-LAMP1-mCherry (red) to directly examine lysosomal morphology after cell fixation. Lysosomes are enlarged in RPE cells under HG conditions compared to LG conditions, suggesting mitophagic flux to lysosomes. There appears to be an active basal mitophagic activity and high lysosome number in RPE cells, which are correlated with its high phagocytic activity to recycle POS in the retina. However, in RPE cells treated with HG, lysosome enlargement and reduced lysosomal enzyme activity occur. Excess lysosome fusion with autophagosomes and/or late endosomes may occur under HG conditions, giving rise to enlarged lysosomes. This process, if prolonged, will cause lysosomal membrane permeabilization (LMP), cathepsin leakage, caspase-1 activation and a decrease in membrane tight junction proteins (Aveleira et al., 2010; Durchfort et al., 2012; Kauppinen et al., 2012; Zhuang et al., 2015).

In conclusion, the question of whether mitophagy is good or bad in various diseases has been raised by several investigators (Burman et al., 2017; Devi et al., 2017; Scheibye-Knudsen et al., 2015). Mitophagy itself is a cellular defense mechanism that is required for preventing cellular stress and maintaining homeostasis and survival. Therefore, an optimal mitophagic rate and balanced biogenesis is critical for preserving mitochondrial numbers, health and bioenergetics. However, under chronic diseases, mitophagy dysregulation, lysosome destabilization and inflammasome activation occur. A slow mitophagy will cause damaged mitochondria to accumulate, whereas excess mitophagic flux will reduce mitochondrial numbers; either process will lead to bioenergetics deficiency, oxidative stress, inflammation and cell death (Devi et al., 2017; Singh et al., 2017). Dysregulation of the mitochondria-lysosome axis in diabetic animal models and human subjects has yet to be investigated. In this study, we demonstrate that HG treatment significantly increases TXNIP levels in RPE cells in both the mitochondrion and cytosol in *in vitro* cultures. TXNIP is a mediator of mitochondrial fragmentation and mitophagic flux to lysosomes in RPE under diabetic conditions, which could be related to events that occur in early DR and diabetic macular edema. Lysosomal enlargement and reductions in acid hydrolase activity may slow down POS phagocytosis and retinal phototransduction in diabetes. Targeting TXNIP and mitochondrial redox proteins may be one way to preserve RPE function in DR.

## MATERIALS AND METHODS

### Cell culture

A human retinal pigment epithelial cell line, ARPE-19, was purchased from ATCC (Cat# 2302), whereas HRPE cells were obtained from LONZA (Walkersville, USA, Cat# 00194987). The cells were maintained in DMEM:F12 medium (1:1 ratio) containing LG, 10% serum, and 1% streptomycin and penicillin. For experiments, at ∼80% confluence, the medium was changed to 2% serum overnight. Then, RPE cells were cultured either in LG (5.5 mM) or HG (25 mM) conditions for 5 days, as described previously (Devi et al., 2012, 2017). When the antioxidant NAC or Amlx, an inhibitor of the protein kinase TBK1, were included in the media; they were added 24 h before ending the experiments.

Transfection of TXNIP shRNA clones in pcDNA3.1 plasmids (Cat# CBGT J0909-1, Creative Biogene, Shirley, USA) were performed with Lipofectamine 3000 (Cat# L3000008) using Opti-MEM Medium (Cat# 31985070) from Thermo Fisher Scientific according to the instructions from the manufacturer. Four different shTXNIP RNAs - #1, 2, 3 and 4 in pcDNA3.1 were initially tested. Among these, a combination of 3+4 gave ∼70-80% knockdown of TXNIP in ARPE-19 cells. Stably-expressing shTXNIP3+4 cells were selected using G417 antibiotic (Life Technologies) and they were used in this study. A scramble RNA containing the pcDNA3.1 plasmid was transfected in ARPE-19 cells as a control.

DMEM (Cat# 10-014-CM) was purchased from Mediatech Inc. (Manassas, USA), F12 Ham's was purchased from HyClone (Cat# SH30026.01), and serum (Cat# MT35010CV) was purchased from Corning (Corning, USA). Antibiotics and trypsin were purchased from HyClone. TXNIP antibodies were obtained from MBL International (Woburn, USA) and Cell Signaling Technology (Danvers, USA). For immunofluorescence, ProLong Gold antifade reagent with DAPI (mounting medium, Cat# P36935) was obtained from Molecular Probes. Slides and coverslips were purchased from Thermo Fisher Scientific. A complete list of reagents, antibodies, PCR primers and their sources are provided in Tables S1–S5.

### Western blotting, qPCR and cell assays

These methods were, aside from minor changes, performed as described previously (Devi et al., 2012, 2013, 2017). For total protein extraction and western blotting, RIPA buffer was used as were precast polyacrylamide gels from BioRad. ImageJ was used to quantitate the blots. Trizol (Ambion) was used to isolate total RNA whereas the iScript™ cDNA Synthesis Kit and SYBR Green reagent (BioRad, Hercules, USA) were used for cDNA synthesis and qPCR detection. The MitoProbe™ JC-1 Assay Kit (Cat# M34152) and ATP Determination Kit (Cat# A22066) were purchased from Thermo Fisher Scientific and used according to their instructions. Cell viability was measured using a fluorescence plate reader and the LIVE/DEAD™ Viability/Cytotoxicity Kit for mammalian cells (Cat# L3224, Invitrogen). For the caspase-1 and cathepsin L assays, the FAM-FLICA^®^ Caspase-1 Assay Kit and Magic Red Cathepsin L Assay Kit, respectively, were purchased from Immunohistochemistry Technologies (Bloomington, USA).

### Mitochondrial fraction isolation

Mitochondrial fractions were isolated to detect mitochondrial proteins using a mitochondria isolation kit for cell cultures (Catalog: 89874, Option B method) from Life Technologies, Thermo Fisher Scientific, according to the manufacturer's instructions. The cytosolic fraction from the mitochondrial preparation was further concentrated using a Microcon-10 kDa Centrifugal Filter Unit with Ultracel-10 membrane (Millipore) to detect cytosolic proteins. Protein was estimated by the Bradford assay and 20–30 µg of protein were used for western blotting. The purity of the mitochondrial fractions was established by the absence of cytosolic or nuclear proteins, LDH and Lamin B1, respectively, as described before (Devi et al., 2017).

### Live-cell imaging

For the live-cell imaging of mt-Keima, ARPE-19 or HRPE cells were cultured in six-well plates. The cells were maintained under LG or HG conditions for 5 days while 2 µl of an adenovirus encoding mt-Keima (Ad-CMV-mt-Keima) with a 4.8×10^10^ PFU/ml GC titer (Vector Biosystems, Inc., Malvern, USA) was transduced in the last 3 days before capturing the images. When CCCP was used to induce mitophagy, it was present for 24 h, whereas inhibitors were added 4 h before capturing the images. The media was changed to HBSS solution without phenol. A Zeiss LMS 780 Confocal Microscope (Germany) fitted with a CO_2_ chamber and live-cell imaging platform was used to capture images in triplicate cultures. We used a W-Plan-Apochromat 63X/1.0NA objective. For the green (mt-Keima in mitochondria) signal we used a 458-nm Argon laser at 5% laser power and our emission collection window was set from 570–694 nm. For the red (mt-Keima in lysosomes) signal we used a 561-nm HeNe laser at 5% laser power and the emission collection window was similar to that of the green signal (570–694 nm). We imaged at 1 AU (airy unit), giving us an optical section thickness of 0.7 µm. In each experiment, all images were captured within 1–1.30 h. Images were analyzed using ZEN 2.3 lite software and compiled in Adobe Photoshop. For quantitation of the red and green signals, the images were converted to black and white and 10–15 cells from three separate images were analyzed by ImageJ software.

### Fixed-cell imaging

For imaging fixed cells, ARPE-19 or HRPE cells were cultured with 2 ml of DMEM:F12 medium in six-well plates, three containing sterile coverslips in each well. Media were changed every 2 days. Similar to live-cell imaging conditions, LG and HG conditions were maintained for 5 days while 2 µl of an adenovirus encoding human LAMP1-mCherry (Ad-CMV-LAMP1-mCherry) with a 4.3×10^10^ PFU/ml GC titer (Vector Biosystems, Inc., Malvern, USA) was added for 3 days. In addition, when present, 4 µl of mt-GFP (CellLight™ Mitochondria-GFP, BacMam 2.0, Invitrogen) was added to the medium for 24 h. Cells were fixed and mounted with DAPI or Hoechst to stain nuclei. A Zeiss LMS 780 Confocal Microscope was used to capture multiple images in triplicate. We used the Plan-Apochromat 63X/1.4NA objective. For the blue signal, we used a 405 nm Diode laser at 10% laser power and our emission collection window was from 415–470 nm. For the mt-GFP, we used a 488-nm Argon laser at 6% power and our emission collection window was from 499–552 nm. For the red (LAMP1-mCherry), we used a 561-nm HeNe laser at 5% power and our emission collection window was from 575–659 nm. We imaged at 1 AU, but this objective gave us an optical section thickness of 1.2 µm. Images were analyzed using ZEN 2.3 lite software and compiled in Adobe Photoshop. The diameters of 15 random lysosomes from three separate images were measured using the ZEN 2.3 lite software and analyzed.

### Statistical analysis

The results are expressed as the mean±s.e.m. of the indicated number of experiments. Comparisons between two sets of experiments were analyzed using the unpaired two-tailed *t*-test, whereas one-way ANOVA followed by the Bonferroni post-hoc test was used to determine differences among means in multiple sets of experiments. A *P*-value of <0.05 was considered to be statistically significant.

## Supplementary Material

Supplementary information
