# Correction: TXNIP mediates high glucose-induced mitophagic flux and lysosome enlargement in human retinal pigment epithelial cells

**DOI:** 10.1242/bio.059489

**Published:** 2022-07-29

**Authors:** Takhellambam S. Devi, Thangal Yumnamcha, Fayi Yao, Mallika Somayajulu, Renu A. Kowluru, Lalit P. Singh

There was an error published in *Biology Open* (2019) **8**, bio038521 (doi:10.1242/bio.038521).

In Fig. 7, an image in panel A, LG ScrRNA was erroneously represented again in panel C, LG shTXNIP.

**Fig. 7:**
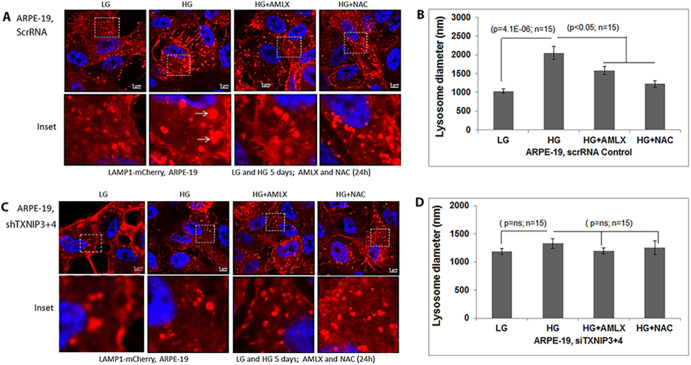
**(corrected). TXNIP knockdown prevents lysosome enlargement in ARPE-19 cells.** (A) In ScrRNA control ARPE-19 cells, HG (5 days) induces lysosome enlargement (LAMP1-mCherry) compared to LG, which is reduced by Amlx (1 μM) and NAC (5 mM). Ad-CMV-LAMP1-mCherry was transduced for 3 days while Amlx and NAC were added 24 h before taking the images. Arrows show enlarged lysosomes under HG. (B) Quantitation of lysosome sizes in scRNA ARPE-19 cells with HG in the absence or presence of Amlx and NAC. Significant lysosome size increase is seen with HG but not in the presence of Amlx or NAC. (C,D) The effect of HG on lysosome enlargement is absent in shTXNIP3+4 ARPE-19 cells, indicating a role for TXNIP in HG-induced lysosome enlargement in ARPE-19 cells. A representative image of n=3 is shown.

The corrected and original figures are shown below. Both the online full-text and PDF versions of the article have been updated. The authors apologise to readers for this error, which does not impact the results or conclusions of this paper.

**Fig. 7:**
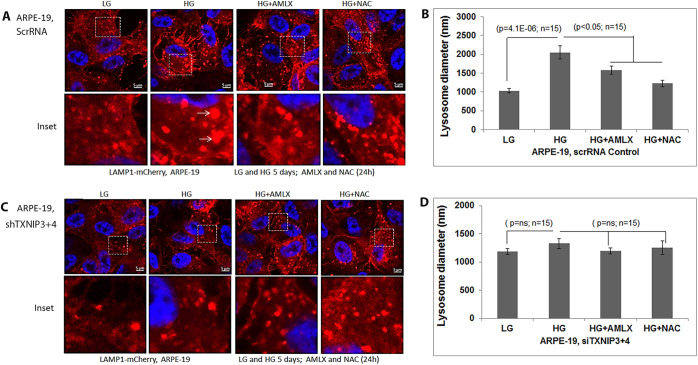
**(original). TXNIP knockdown prevents lysosome enlargement in ARPE-19 cells.** (A) In ScrRNA control ARPE-19 cells, HG (5 days) induces lysosome enlargement (LAMP1-mCherry) compared to LG, which is reduced by Amlx (1 μM) and NAC (5 mM). Ad-CMV-LAMP1-mCherry was transduced for 3 days while Amlx and NAC were added 24 h before taking the images. Arrows show enlarged lysosomes under HG. (B) Quantitation of lysosome sizes in scRNA ARPE-19 cells with HG in the absence or presence of Amlx and NAC. Significant lysosome size increase is seen with HG but not in the presence of Amlx or NAC. (C,D) The effect of HG on lysosome enlargement is absent in shTXNIP3+4 ARPE-19 cells, indicating a role for TXNIP in HG-induced lysosome enlargement in ARPE-19 cells. A representative image of n=3 is shown.

