# Supplementary material for: TXNIP mediates high glucose-induced mitophagic flux and lysosome enlargement in human retinal pigment epithelial cells
Source: Biol Open. 2019 Apr 25;8(4):bio038521. doi: 10.1242/bio.038521 (PMC6503994; doi:10.1242/bio.038521)
Supplement: Supplementary information [file biolopen-8-038521-s1.pdf]

Supplementary Information

Table S1. Chemicals

Name	Company name	Cat #
Amlexanox	Sigma	SML0517
CCCP	Sigma	C2759
NAC	Sigma	A9165
Glucose	Sigma	G8270
Mannitol	Sigma	M4125
Azaserine	Sigma	A-1164
Bafilomycin A	Sigma	B-1793
LeuLueMethy-ester	Sigma	L-7393
Akt inhibitor VIII	Millipore	124018
Hydrogen peroxide	Sigma	95321

Table S2. Antibodies: Primary antibodies for Western blotting

Name	Dilution	Species	Company	Cat #
TXNIP	1:1000	mouse	MBL	K0205-3
TXNIP	1:1000	Rabbit	Cell Signalling	14714S
Actin	1:2000	Goat	Santa Cruz	Sc-1616
Trx2	1:500	Rabbit	Santa Cruz	sc-50336
Trx2	1:1000	Rabbit	Cell Signaling	14907S
Optineurin	1:500	Mouse	Santa Cruz	sc-166576
P62/SQSTM1	1:1000	Rabbit	ThermoFisher	PA5-20839
LC3B	1:3000	Rabbit	Pierce	L10382

SOD1	1:1000	Rabbit	ProteinTech	10269-1-AP
Trx1	1:500	Rabbit	Santa Cruz	SC-20146
Trx1	1:1000	Rabbit	Cell Signaling	2429S
LDH-A	1:1000	Rabbit	Invitrogen	PA5-27406
Lamin B1	1:1000	Rabbit	Cell Signaling	12586

Table S3. Secondary Antibodies: for Western blotting

**Antibody**	**Source**	**Cat#**	**Dilution**
Anti-mouse 2°ry antibody HRP	Sigma	A5278	1:5000
Anti-rabbit 2°ry antibody HRP	Sigma	A6154	1:5000
Anti-goat 2°ry antibody HRP	Santa Cruz	sc-2020	1:5000

Table S4. Immunofluorescence:

**Antibody**		**Source**	**Cat#**	**Dilution**
LAMP2A	mouse	Thermo Scientific	MA5-17861	1:50
LAMP2A	rabbit	Protein Tech	10397-1-AP	1:50
COXIV	mouse	Life Technologies	A-21348	1:200
TrxR2	mouse	Santa Cruz	sc-166259	1:200
Anti-mouse 2°ry Ab, Alexa Fluor 488		Jackson	115-545-146	1:400
Anti-mouse 2°ry Ab, Alexa Fluor 594		Jackson	115-585-146	1:400
Anti-rabbit 2°ry Ab, Alexa Fluor 488		Life Technologies	A-110 34	1:400
Anti-rabbit 2°ry Ab, Alexa Fluor 594		Jackson	111-585-176	1:400

Biology Open • Supplementary information

Table S5. QPCR Primers: (Human)

Gene name	Conc.	Company	Cat#
TXNIP	400 nM	Qiagen	PPH02855A
Trx1(TXN)	400 nM	Qiagen	PPH18986C
Trx2 (TXN2)	400 nM	Qiagen	PPH06073A
Actin-β	400 nM	Qiagen	PPH00073G
Caspase 1	400 nM	Qiagen	PPH00105C
NLRP3	400 nM	Qiagen	PPH13170A
IL-1β	400 nM	Qiagen	PPH00171C

Supplementary Figures

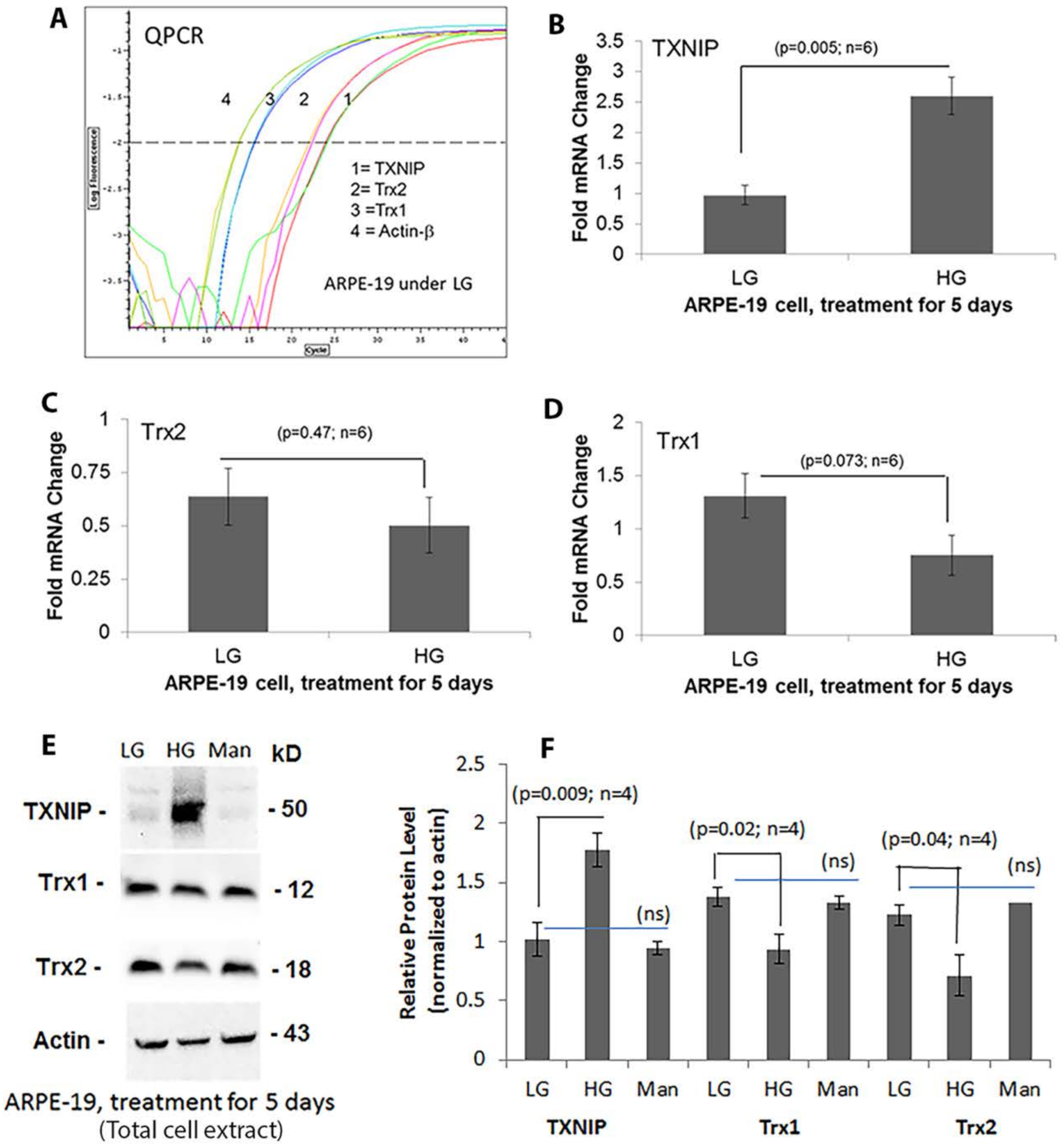

Figure S1. High glucose induces TXNIP expression in ARPE-19 cells in mRNA and protein levels. **(A)** Ct profiles of TXNIP, Trx2, Trx1 and β-actin in ARPE-19 cells in LG. TXNIP mRNA level is lowest followed by Trx2. Trx1 and β-actin levels are higher. **(B)** HG (25 mM) significantly increases TXNIP mRNA expression in ARPE-19 cells when compared to LG (5.5 mM). **(C)** Trx2 and **(D)** Trx1 mRNA levels are downregulated marginally although statistically not significant ($p>0.05$). **(E)** HG also increases TXNIP protein levels significantly but not by a similar osmolar concentration of mannitol at 25 mM **(F)**, indicating that the increase in TXNIP level by HG is not due to osmolar effects. Conversely, Trx1 and Trx2 are significantly reduced by HG but not by mannitol. A representative of n=3 is shown.

Biology Open • Supplementary information

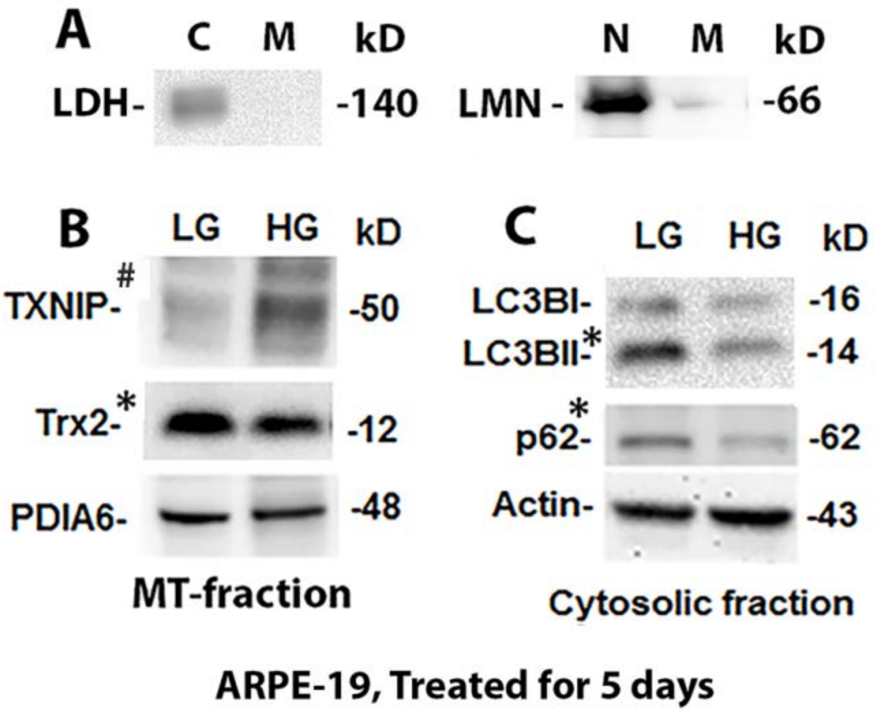

Figure S2. High glucose-induced TXNIP expression in mitochondria is associated with decreases in mitochondrial redox protein Trx2 and autophagic/mitophagic markers in ARPE-19 cells. **(A)** Cytosolic, mitochondrial and nuclear fractions are isolated. The purity of mito-fraction is established by the absence of cytosolic lactate dehydrogenase (LDH) and nuclear lamin b1 (LMN). **(B)** After treatment with LG and HG for 5 days, mitochondrial fractions are subjected to Western blotting for TXNIP and Trx2. HG increases TXNIP significantly (#, $p=0.04$; $n=4$) compared to LG while HG significantly reduces Trx2 (*, $p<0.05$, $n=4$) when compared to LG. Mitochondrial protein disulfide isomerase A6 PDIA6) is not significantly changed, so we use it to normalize TXNIP and Trx2 levels. **(C)** The cytosolic fractions were analyzed for autophagy/mitophagy markers, LC3BI and II and p62/SQSTM1 on Western blots. LC3BII and p62/SQSTM1 are significantly reduced by HG (*, $p<0.05$, $n=4$) when normalized to actin.

Biology Open • Supplementary information

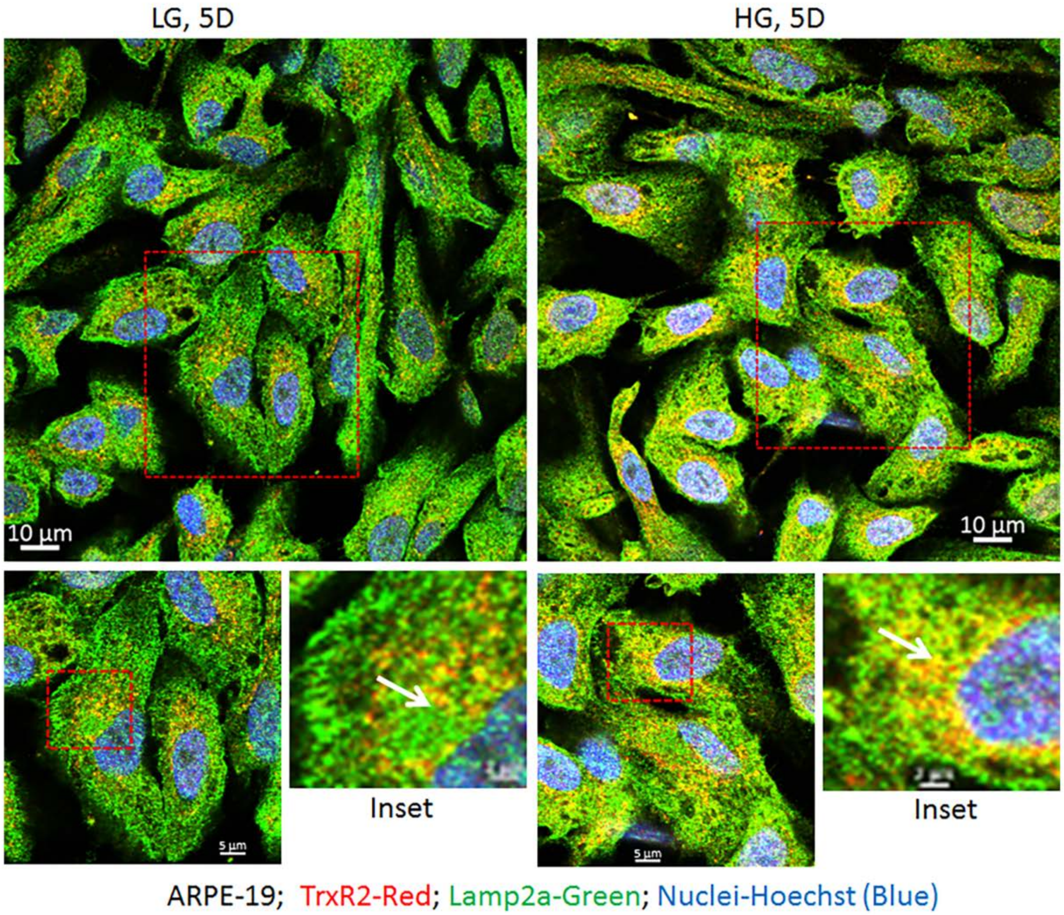

S3. Mitophagy is induced in ARPE-19 by high glucose. Supporting the observation in Figure 1E for mitophagy induction in ARPE-19 cells by high glucose, we also observed an increase in co-localization of the lysosomal membrane protein LAMP2A with mitochondrial thioredoxin reductase 2 (TrxR2) under high glucose (HG, 25 mM) when compared with low glucose (LG, 5 mM, inset arrows) indicating mitophagic flux to lysosomes. Fixed cell images were captured in a Zeiss confocal microscope at x630 magnification. A representative image of n=3 is shown.

Biology Open • Supplementary information

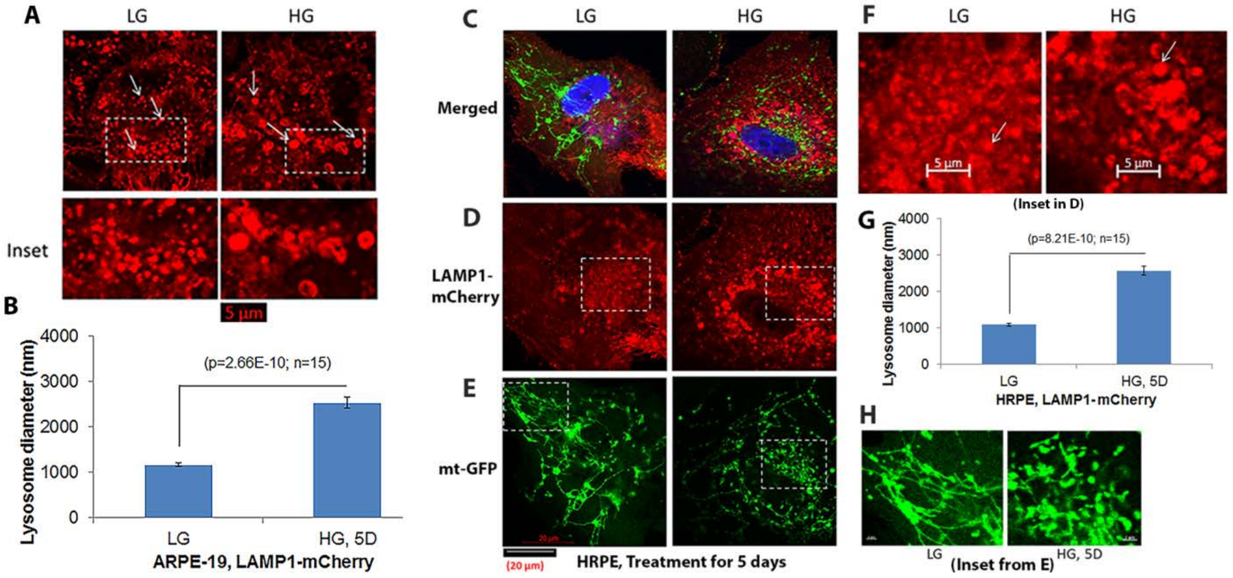

Figure S4. High glucose induces mitochondrial fragmentation and lysosome enlargement in human RPE cells. (A-B) Ad-CMV-LAMP1-mCherry targeting to lysosomes is transiently transfected in ARPE-19 cells and they are treated with HG or LG for 5 days. HG significantly increases lysosome sizes in ARPE-19 cells. **(C)** Furthermore, mitochondria-targeted GFP, mt-GFP and LAMP1-mCherry are co-transduced in primary HRPE cells. **(D, F, G)** Significant lysosomal enlargement is seen with LAMP1-mCherry expression in HRPE under HG (5 days) when compared to LG. **(E, H)** HG (treatment for 5 days) causes mitochondrial fragmentation and when compared to LG. Images are captured in a Zeiss confocal microscope at x630 magnification. A representative image of n=3 is shown.

Biology Open • Supplementary information

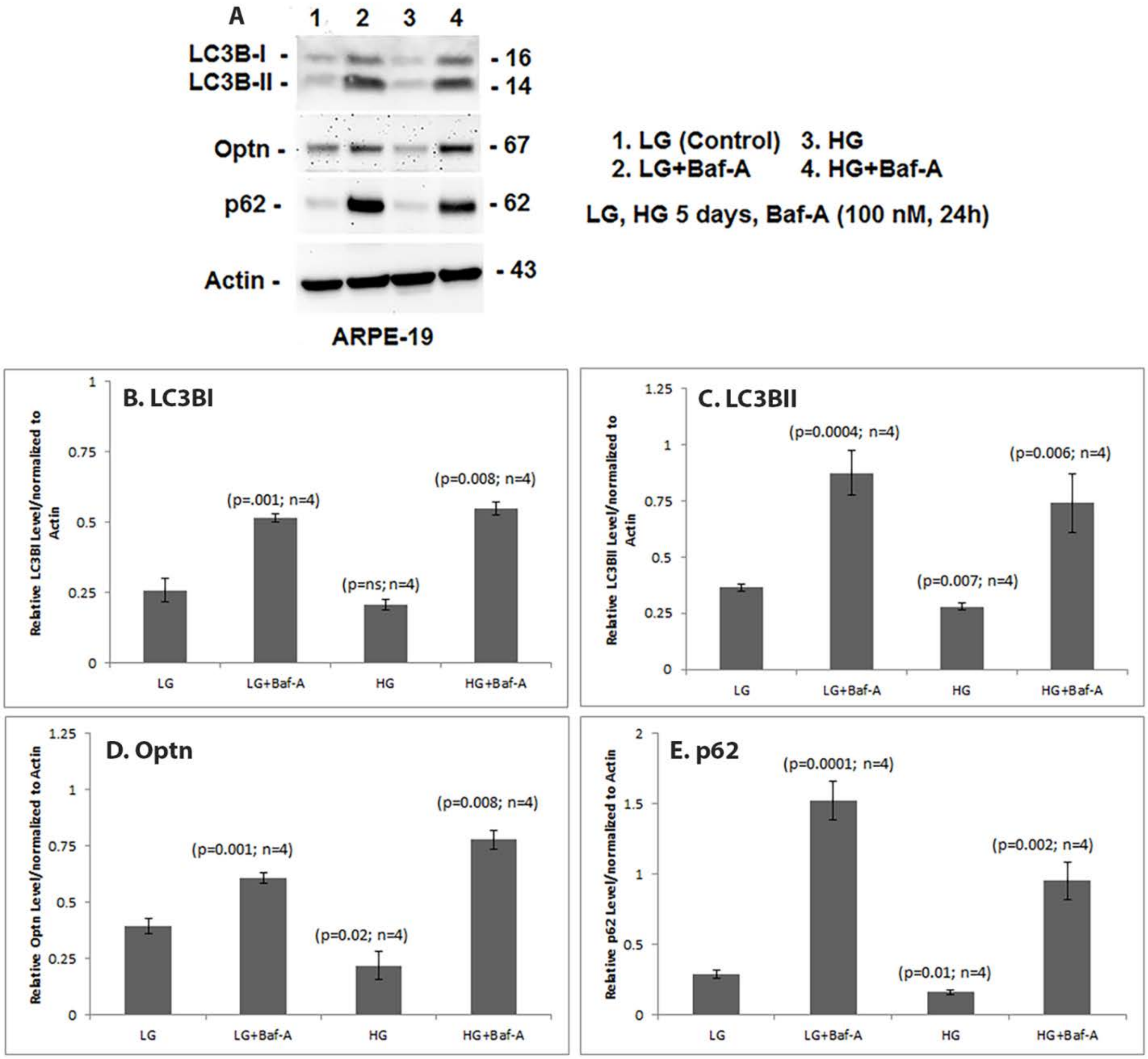

Figure S5. Bafilomycin A inhibits high glucose-induced mitophagic flux in ARPE-19. Bafiloycine A (Baf-A) is an inhibitor of lysosomal membrane H+-ATPases (V-ATPases), therefore, inhibits lysosome and autophagosome fusion.. Therefore, degradation autophagic or mitophagic proteins is blocked leading to their accumulation. Here we show that Baf-A increases the level of mitophagic adaptors Optn and p62 and double membrane protein LC3B both in LG and HG conditions (lanes 2 and 3). Conversely, in the absence of Baf-A, a decrease in optn and p62 level is seen, which suggests their flux to lysosomes and degradation. Furthermore, HG significantly reduces both Optn and p62 levels indicating a mitophagic flux.

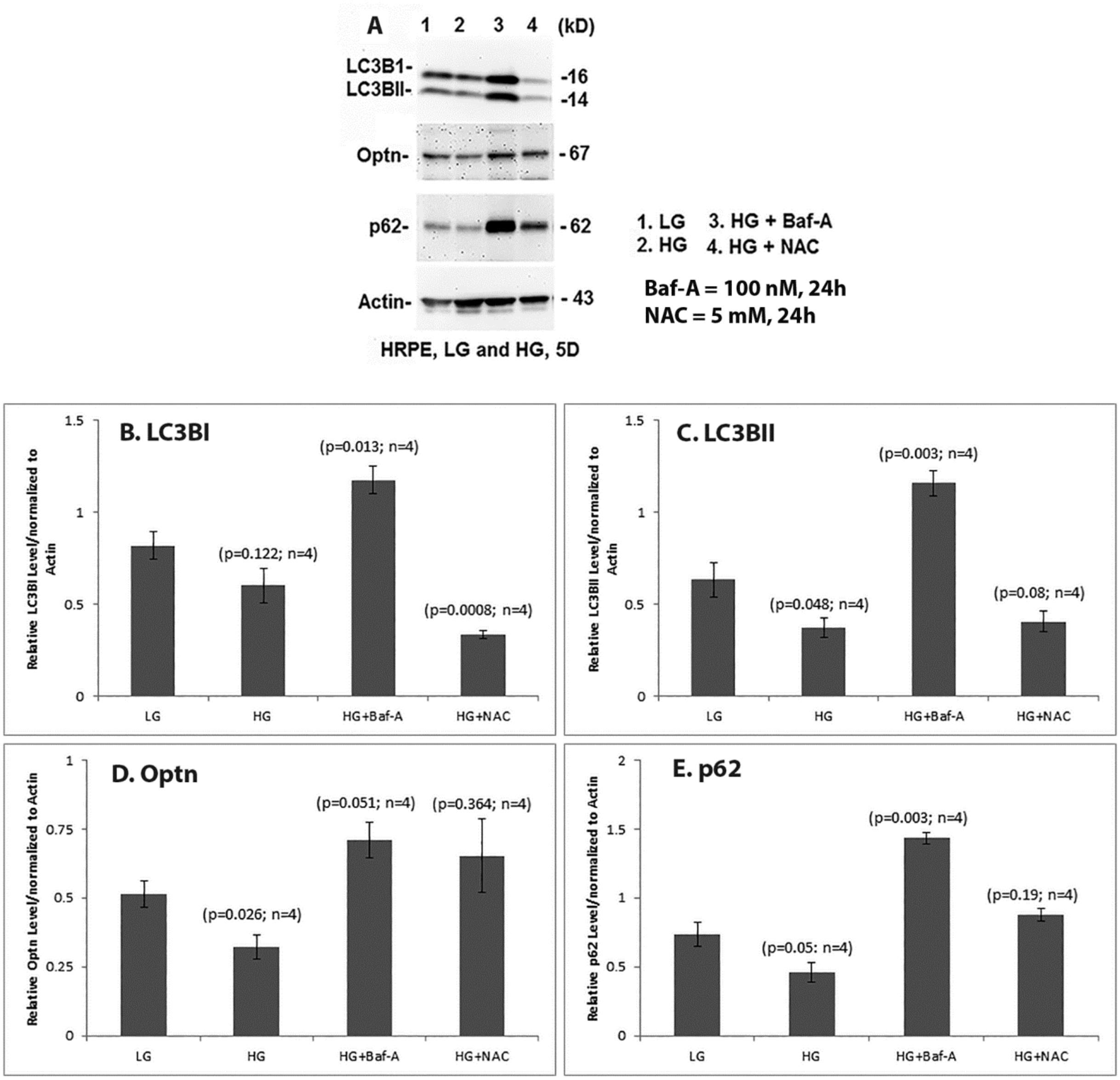

Figure S6. Bafilomycin A inhibits high glucose-induced mitophagic flux in HRPE cells. HG decreases mitophagy adaptors Optin and p62 as well as LC3B levels than in LG, suggesting a mitophagic flux and lysosomal degradation. Upon Baf-A addition, which inhibits lysosome-autophagosome fusion, their levels are increased suggesting a blockade of the lysosomal degradation.

Biology Open • Supplementary information

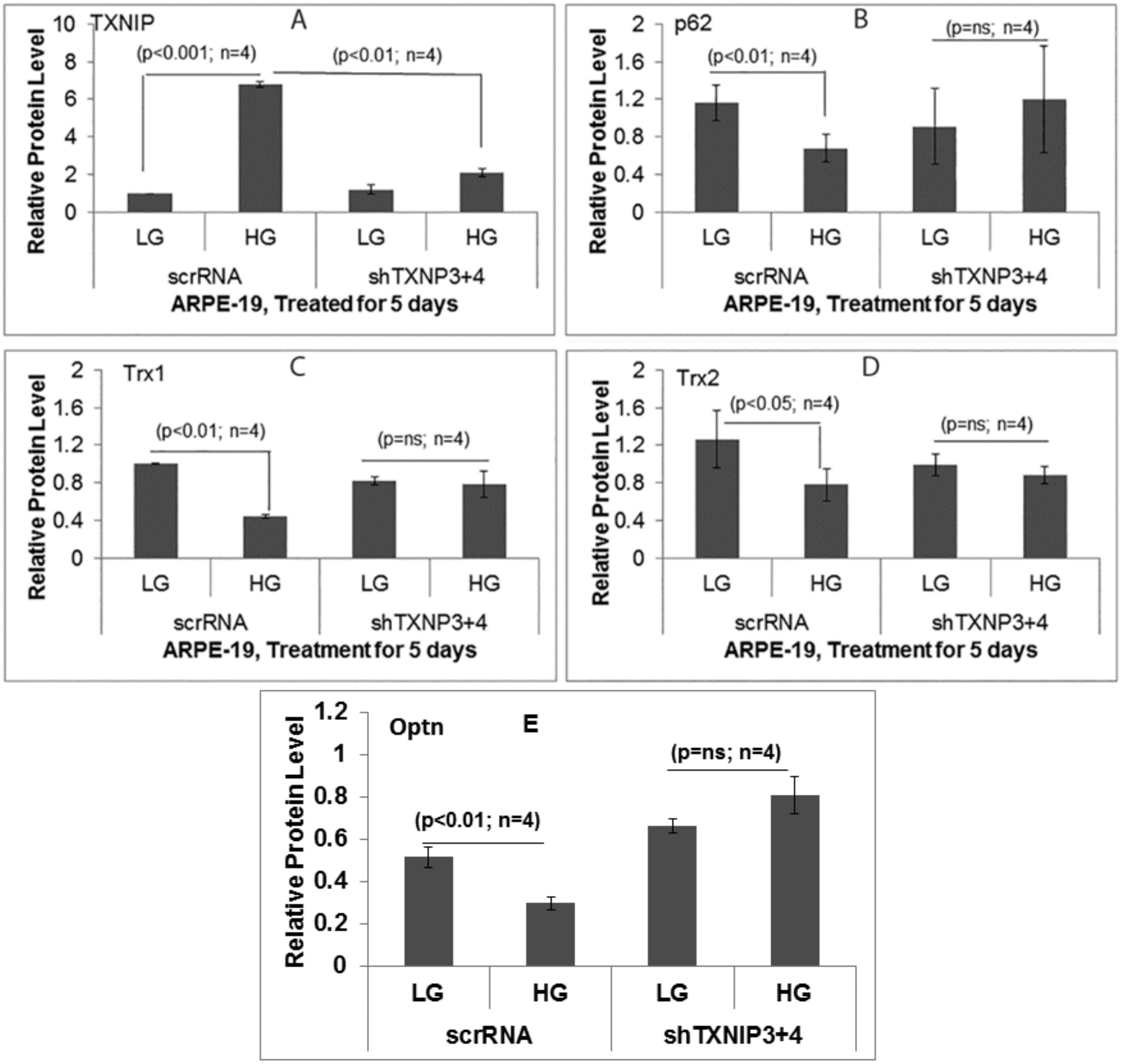

Figure S7. TXNIP knockdown by shRNA reversed Trx1, Trx2 and p62/SQSTRM1 downregulation by HG in ARPE-19 Cells. Densitormetric data analysis of Figure 5D Western blots. **(A)** TXNIP is significantly induced by HG in scrRNA control ARPE-19 cells while it is blunted in shTXNIP3+4. TXNIP shRNA reverses HG- induced decreases in **(B)** p62/SQSTM1, **(C)** Trx1, **(D)** Trx2 and **(E)** Optn in scrRNA control transfected ARPE-19 cells.

Biology Open • Supplementary information

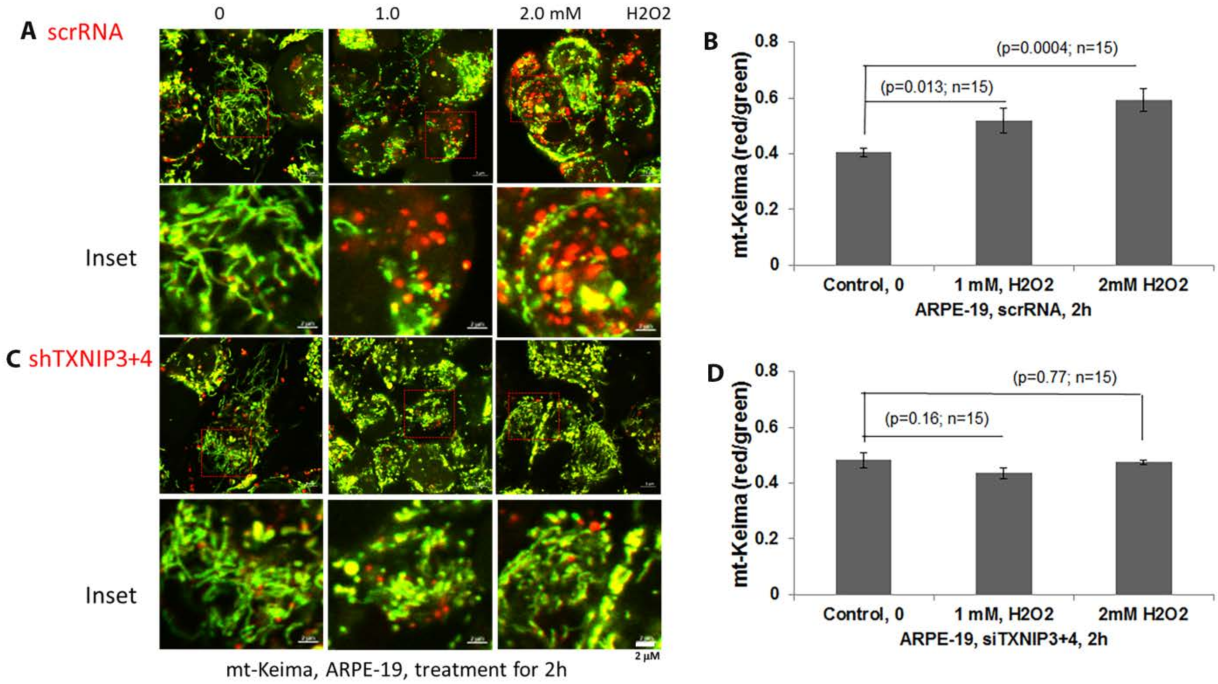

Figure S8. Hydrogen peroxide (H_2O_2)-induced mitophagy is prevented by TXNIP knockdown in ARPE-19 cells. (A-B) H_2O_2 treatment (1 mM and 2 mM) in ARPE-19 cells for 2 h induces mitophagic flux as indicated by the presence of red mt-Keima in lysosomes and a densitometric analysis of the mt-Keima red/green. **(C)** Green mt-Keima predominates in shTXNIP3+4 cells both under 1 mM and 2 mM H2O2 and **(D)** the mt-Keima red/green ratio shows no significant difference between control and H2O2 treated shTXNIP3+4 APRE1-9 cells. Images were captured in a Zeiss confocal microscope at x630 magnification. A representative image of n=3 is shown.

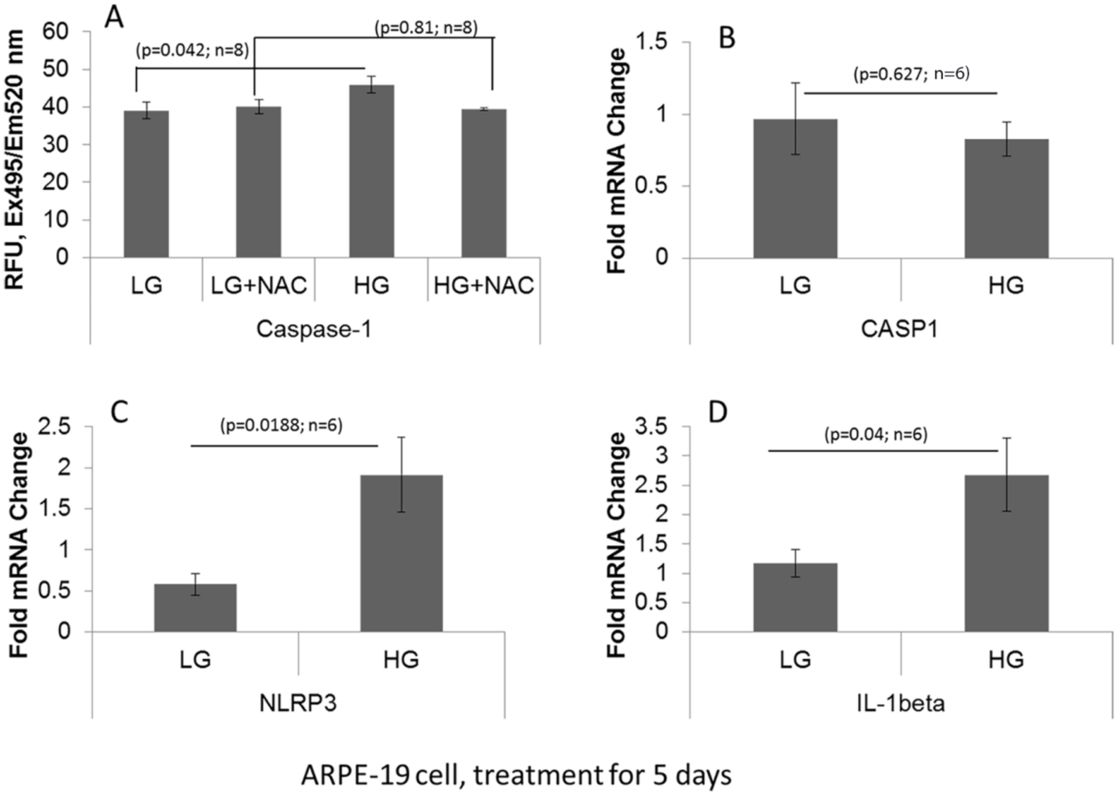

Figure S9. HG increases caspase-1 activity in ARPE-19 cells. (A) Treatment of ARPE-19 cells with HG for 5 days increases caspase-1 activity significantly ($p<0.05$) when compared to LG. NAC (5 mM) prevents HG-induced caspase-1 activition. **(B)** The mRNA expression of caspase-1 is not changed by HG while that of **(C)** NLRP3 and **(D)** IL-1β were significantly increased by HG when compared to LG.

Biology Open • Supplementary information

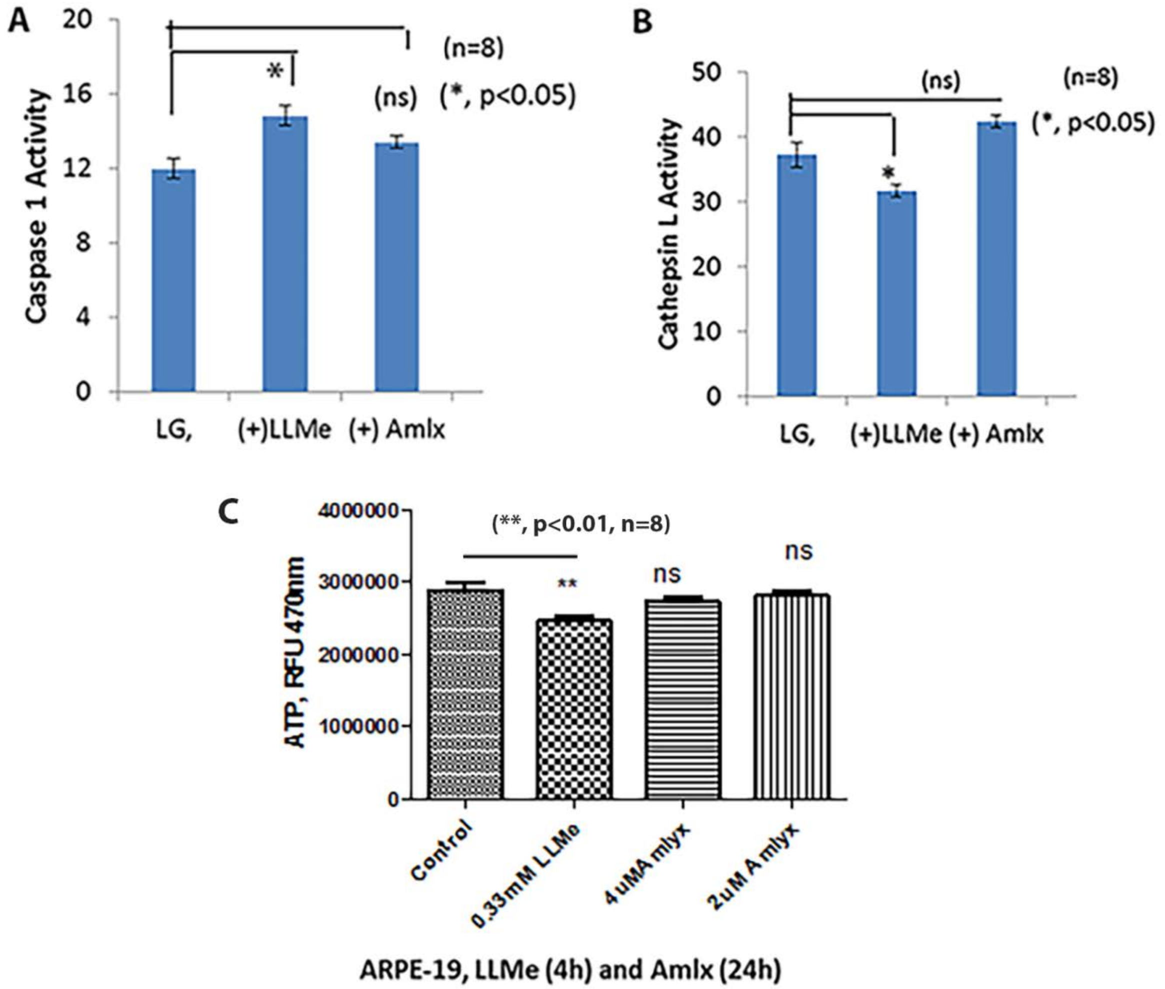

Figure S10. LLMe increases caspase 1 activity and decreases Cathepsin L activity in ARPE-19 cells. **(A)** Treatment for ARPE-19 cells with LLMe (0.33 mM), which causes lysosomal membrane permeability (LMP), for 4 h increases caspase 1 activity significantly ($p<0.05$; n=8) than in LG control. However, Amlx (1 μM), which inhibits TBK1/IKKε kinases, has no effect on the caspase 1 activity. **(B)** Conversely, lysosomal enzyme Cathepsin L activity is reduced by LLMe ($p<0.05$, n=8), while Amlx (1 μM) maintains cathepsin L activity. **(C)** LLMe also reduces ATP level in ARPE-19 cells while Amlx has no effect on ATP. These results suggest that Amlx may be a potential drug to inhibit mitophagic flux and lysosomal damage in retinal neurodegeneration and diabetic retinopathy.

Biology Open • Supplementary information
